# Bioinspired lipoproteins-mediated photothermia remodels tumor stroma to improve cancer cell accessibility of second nanoparticles

**DOI:** 10.1038/s41467-019-11235-4

**Published:** 2019-07-25

**Authors:** Tao Tan, Haiyan Hu, Hong Wang, Jie Li, Zhiwan Wang, Jing Wang, Siling Wang, Zhiwen Zhang, Yaping Li

**Affiliations:** 1 State key Laboratory of Drug Research & Center of Pharmaceutics, Shanghai Institute of Materia Medica, Chinese Academy of Sciences, Shanghai, 201203 China; 2 School of Pharmacy, Shenyang Pharmaceutical University, Benxi, 117000 Liaoning China; 3 School of Pharmacy, Yantai University, Yantai, 264005 Shandong China; 4 Yantai Key Laboratory of Nanomedicine & Advanced Preparations, Yantai Institute of Materia Medica, Shandong, 264000 China

**Keywords:** Drug delivery, Cancer microenvironment, Bioinspired materials, Drug delivery

## Abstract

The tumor stromal microenvironments (TSM) including stromal cells and extracellular matrix (ECM) form an abominable barrier hampering nanoparticles accessibility to cancer cells, significantly compromising their antitumor effects. Herein, we report a bioinspired lipoprotein (bLP) that can induce efficient photothermia to remodel TSM and improve second bLP accessibility to cancer cells for antitumor therapy. The multiple stromal cells and ECM components in TSM are remarkably disrupted by bLP-mediated photothermal effects, which cause a 4.27-fold enhancement of second bLP accumulation in tumor, deep penetration in whole tumor mass and 27.0-fold increase of accessibility to cancer cells. Of note, this bLP-mediated TSM-remodeling to enhance cancer cell accessibility (TECA) strategy produces an eminent suppression of tumor growth and results in a 97.4% inhibition of lung metastasis, which is superior to the counterpart liposomes. The bLP-mediated TECA strategy provides deeper insights into enhancing nanoparticle accessibility to cancer cells for antitumor therapy.

## Abstract (web summary)

The stromal cells and extracellular matrix hamper nanoparticle access to cancer cells and their anti-cancer efficacy. Here, the authors report a bioinspired lipoprotein (bLP) for photothermal remodelling of tumour stroma and show this to improve subsequent bLP accessibility to cancer cells.

## Introduction

Nanosystems can preferentially deliver various therapeutic agents to tumors for cancer therapy^1–3^. To date, several nanosystems of doxorubicin (Doxil, Caelyx, and Myocet), irinotecan (Onivyde), paclitaxel (Abraxane), and vincristine (Marqibo) have been approved for clinical cancer indications, and many other nanosystems are undergoing clinical trials^4^. However, these nanosystems have shown only moderate therapeutic benefits in clinic^5^, which are presumably due to their poor accessibility to cancer cells in tumor^6,7^. Nanosized particles can be passively accumulated at tumor sites based on the leakiness of tumor vasculature, but only a small fractions (often less than 5%) can reach the tumor sites in most cases^8,9^. Worse still, the rapid proliferation of cancer cells forces blood vessel apart and makes them distant (usually > 100 µm) from tumor vasculature^7^. Most nanoparticles only diffuse more than one or two cell layers away from the blood vessels, and are poorly accessed to the cancer cells in tumor, thereby compromising their therapeutic efficacy^6,10^.

With improved understanding of tumor biology, cancer cells are co-evolved with stromal cells including cancer-associated fibroblasts (CAF) or tumor-associated macrophages (TAM) to promote their progression and metastasis^11–14^. CAF and TAM are the most abundant stroma cells in tumor tissues. CAF can produce a large variety of extracellular matrix (ECM) components, including collagen, fibronectin, and hyaluronan, etc.^13,15^. By contrast, TAM are believed to shape the tumor stroma by producing proteolytic enzymes and matrix-associated proteins, and are also pivotal constructors of tumor collagenous matrix through regulation of CAF^16^. These stroma cells and multiple ECM components form the tumor stromal microenvironments (TSM) barriers^13–15^. Nanoparticles that have reached the tumor sites are dominantly hijacked by CAF and TAM, and their permeation in tumor mass is greatly impeded by the dense network of the ECM^17–20^ Conceivably, remodeling the TSM barriers can be a substantial strategy to promote nanoparticles accessibility to cancer cells for anticancer therapy.

**Fig. 1:**
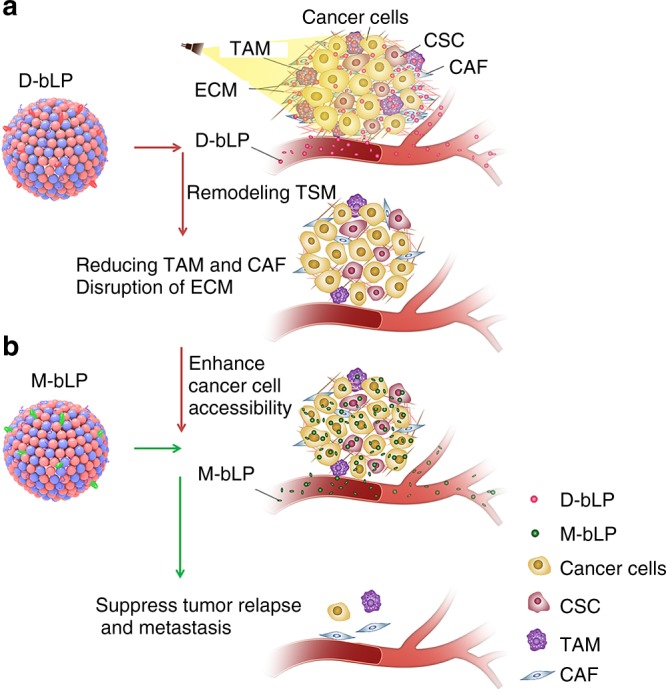
Schematic illustration of D-bLP-mediated photothermal remodeling of tumor stroma to enhance second M-bLP accessibility to cancer cells. **a** D-bLP-mediated photothermal effects cause drastic elimination of stromal cells (e.g., CAF and TAM) and ECM components (e.g., collagen I, fibronectin). **b** D-bLP-mediated TSM remodeling enhances the accumulation and penetration of second M-bLP in tumor, promotes their extravasation from tumor vasculature and accessibility to cancer cells, thus resulting in efficient suppression of tumor relapse and metastasis. CSC cancer stem cells

The photothermal effects that are generated upon near-infrared (NIR) light irradiation exhibit unique advantages in cancer treatments^21–23^. Apart from their effects in damaging cancer cells and increasing vascular permeability in tumors^24,25^, the multifaceted impacts on TSM have been scarcely understood. Lipoproteins, especially the high-density lipoproteins (HDL), are endogenous nanostructured particles composed of diverse proteins (e.g., apolipoprotein A1, ApoA1) and lipids (e.g., phospholipids and cholesterol esters), potentiating them ideal nanoplatform to deliver various imaging or therapeutic agents to tumor^26–36^. Accordingly, we here design a bioinspired lipoprotein (bLP) nanosystem that, respectively, loads photothermal agent of DiOC_18_(7) (DiR) (termed as D-bLP) and anticancer drug of mertansine (termed as M-bLP), aiming at remodeling TSM barriers with D-bLP-mediated photothermia and augmenting the accessibility of second-wave M-bLP to cancer cells in tumor for efficient suppression of tumor relapse and metastasis (Fig. 1).

In this bLP-based TSM remodeling to enhance the cancer cells accessibility (TECA) strategy, the lipophilic DiR is used as an imaging and photothermal agent, while the cytotoxic mertansine is intended as a therapeutic agent for antitumor therapy^21,37,38^. The tumor targeting behavior of D-bLP and counterpart liposomal formulations are investigated in a metastatic 4T1 breast cancer model. The impact of D-bLP-mediated photothermia on stromal cells (e.g., CAF and TAM) and major ECM components (e.g., collagen I and fibronectin) are illustrated. The merit of D-bLP-mediated TSM remodeling on tumor accumulation, penetration, and cancer cell accessibility of second-wave M-bLP are underscored. The therapeutic efficacy is evaluated in two breast cancer models to validate the effectiveness on tumor relapse and metastasis.

## Results

### Characterization of D-bLP and M-bLP

**Fig. 2:**
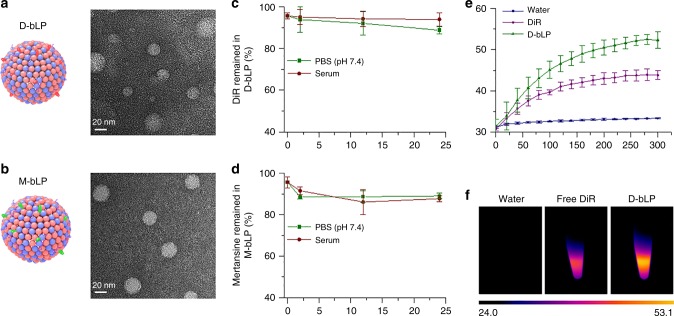
Preparation and characterization of D-bLP and M-bLP. **a** Typical TEM images of D-bLP, scale bar, 20 nm. **b** Typical TEM images of M-bLP, scale bar, 20 nm. **c** Stability of D-bLP in PBS (pH 7.4) and whole FBS, which was characterized by percentage of DiR remained in the D-bLP system (*n* = 3). **d** Stability of M-bLP in PBS (pH 7.4) and whole FBS, which was characterized by percentage of mertansine remained in the M-bLP system (*n* = 3). **e** Temperature changes of water, free DiR, and D-bLP upon their exposure to 808 -nm laser for 5 min (*n* = 3). **f** Typical thermal images of water, free DiR, and D-bLP upon 808 -nm laser irradiation. The data are means ± SD

Inspired by the components and biological properties of HDL, bLP were fabricated with synthetic phospholipids of 1,2-dimyristoyl-sn-glycero-3-phosphatidylcholine (DMPC), 1,2-dioleoyl-sn-glycero-3-phosphoethanolamine (DOPE), and ApoA1-mimetic peptides (sequence: PVLDLFRELLNELLEALKQKLK). The lipophilic DiR and mertansine were, respectively, loaded to prepare the D-bLP (0.5 mg·mL^−1^) and M-bLP (0.05 mg·mL^−1^) for further measurements. The morphologies were visualized under field-emission transmission electronic microscopy (FE-TEM). In the typical FE-TEM images, nanosized spherical particles were readily visualized, but discoidal nanoparticles were rarely detectable (Fig. 2a, b; Supplementary Fig. 1), suggesting that both D-bLP and M-bLP could presumably be spherical nanoparticles. Statistical image analysis showed that the mean diameter was 23.6 ± 4.8 nm for D-bLP and 26.4 ± 5.2 nm for M-bLP. The exact compositions of DiR, mertansine, phospholipids, and/or peptides/proteins in D-bLP and M-bLP were measured in comparison with natural mature HDL from human plasma (Supplementary Table 1). By analyzing the particle size, morphology, and compositions of these formulations with natural mature HDL, the nanosystems of D-bLP and M-bLP could be termed as bioinspired lipoproteins. The difference between bLP and natural mature HDL in particle size and zeta potential values could be ascribed to the involved ingredients of synthetic phospholipids, active therapeutic agents, and mimetic peptides. Considering the efficient tumor targeting ability of the bLP system^30,36,39,40^, D-bLP and M-bLP could be preferentially accumulated at tumor sites to exert the therapeutic efficacy.

The lipophilic DiR was loaded in D-bLP with a drug loading (DL) capacity of 4.44 ± 0.21% (w/w), and encapsulation efficiency (EE) of 95.64 ± 1.51%. Meanwhile, mertansine was incorporated in M-bLP with the DL capacity of 0.464 ± 0.004% (w/w) and EE value of 95.61 ± 2.67%. These results suggested the high encapsulation of DiR and mertansine in these bLP. Then, we investigated the stability of D-bLP and M-bLP in phosphate buffered solution (PBS, pH 7.4) and whole fetal bovine serum (FBS). Following a 24 h of incubation, both DiR and mertansine were slowly released from the D-bLP or M-bLP in these two media. After 24 h of incubation, more than 88% DiR remained in D-bLP, and over 86% mertansine was incorporated in M-bLP (Fig. 2c, d). By contrast, over 90% DiR and mertansine were entrapped in the counterpart formulations of DiR-loaded liposomes (D-Lipo) and mertansine-loaded liposomes (M-Lipo) after 24 h incubation in PBS or FBS (Supplementary Fig. 2). These results suggested the good stability of these nanosystems in the mimicked physiological fluids, and confirmed their feasibility for in vivo delivery.

In addition, the heat-generating ability of D-bLP was determined upon their exposure to an 808 -nm laser at 2.0 W·cm^−2^ for 5 min. In Fig. 2e, f, the temperature could be increased to 52.5 ± 1.2 °C in D-bLP versus 43.9 ± 1.4 °C in free DiR. By contrast, the temperature of counterpart D-Lipo could be elevated to 51.4 ± 1.2 °C, which was comparable with that of D-bLP (Supplementary Fig. 3). These measurements suggested the efficient photothermal effects of D-bLP and D-Lipo.

### In vivo tumor targeting behavior of D-bLP

**Fig. 3:**
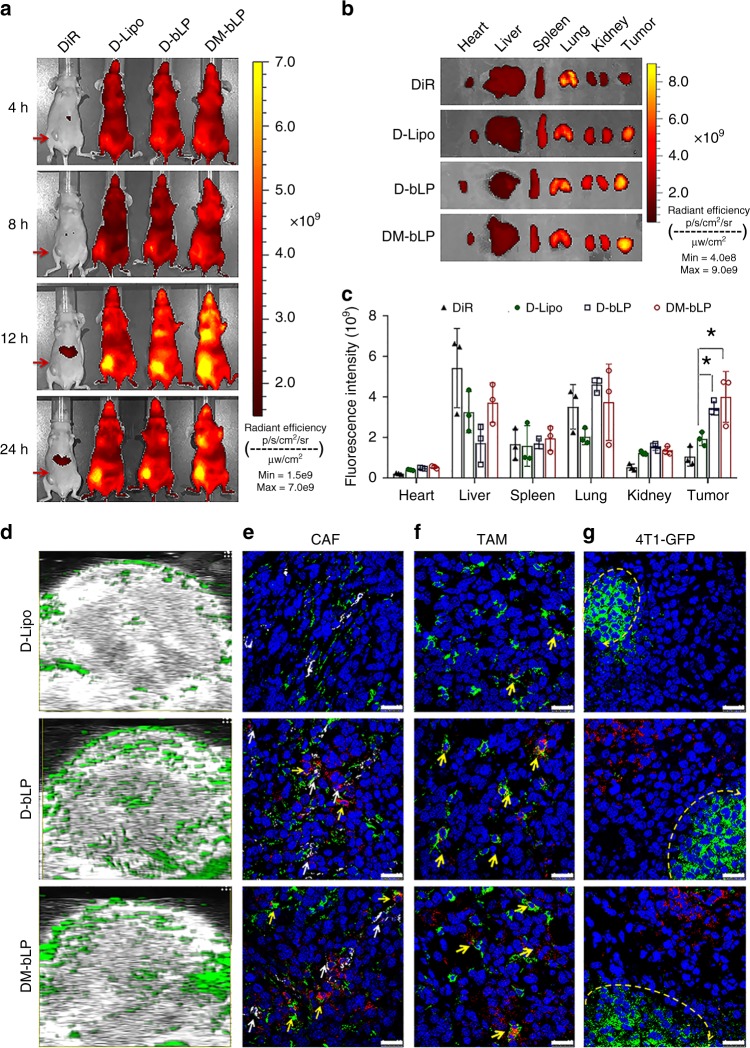
The in vivo tumor targeting and internalization by various cells at tumor sites of the 4T1 breast cancer model. **a** Specific accumulation of free DiR, D-Lipo, D-bLP, and DM-bLP at tumor sites (red arrows) by in vivo imaging system. **b** The ex vivo imaging of each group in major organs. **c** Quantified distribution of each group in major organs (*n* = 3). The data are means ± SD, *p < 0.05 (two-tailed Student’s *t* test). **d** Intratumoral distribution of various nanosystems measured by photoacoustic imaging (green signals in the captured images). **e** Internalization of various nanosystems by CAF at tumor sites. CAF was denoted as cells of α-SMA-positive (green signals) without co-localizing with CD31 (white signals) to avoid the interference of EC. Yellow arrows, CAF; white arrows, EC. **f** Internalization of various nanosystems by TAM at tumor sites. Green signals, TAM; yellow arrows, co-localization of nanosystems with TAM. **g** Internalization of various nanosystems by 4T1-GFP cancer cells at tumor sites. Green signals, 4T1-GFP cancer cells, yellow circle arc, 4T1-GFP cancer cells regions, scale bar, 25 µm

The tumor accumulation of D-bLP and their internalization by various cells at tumor sites were investigated in a metastatic 4T1 cells induced tumor model, which were developed by injecting 4T1 cells to the fourth mammary pad of nude mice. In Fig. 3a, the fluorescence signals from D-bLP, D-Lipo, and DiR-labeled M-bLP (DM-bLP) could be obviously detected at tumor sites at various time points, which were maximized at 12 -h post injection. In tumor (Fig. 3b, c), the fluorescence intensity from D-bLP and DM-bLP was significantly higher than that from D-Lipo (*p* < 0.05) (Fig. 3c). The intratumoral distribution of these nanosystems was determined by photoacoustic imaging (green signals in Fig. 3d). Both D-bLP and DM-bLP could deeply penetrate the tumor mass, while D-Lipo was mainly located in the outer regions of tumor. Therefore, D-bLP and DM-bLP displayed similar in vivo distribution profiles, which exhibited superior tumor accumulation and penetration capacity versus the counterpart D-Lipo. The opening in the tumor ECM is generally <40 nm, and increasing data have confirmed the efficient penetration capacity of small-sized nanoparticles in solid tumors^19,21,39,41,42^. The small particle size of D-bLP and DM-bLP (< 30 nm) may be a crucial contributor to the superior tumor-penetrating capacity. In addition, the ApoA1 mimic peptides in bLP may provide an opportunity to simulate the nanostructure and biological properties of HDL to enhance the tumor targeting ability^30–33^.

Compelling evidence has revealed the preferential accumulation of nanoparticles in tumor after intravenous dosing^3,4,19,43^. We then investigated the uptake of bLP by various cells in tumor using laser confocal scanning microscopy (LCSM) (Fig. 3e–g). In tumor biopsy, versatile cells of TAM, CAF, endothelial cells (EC), cancer cells, and immune cells are heterogeneously distributed^11,13,44–46^ The captured images showed that 4T1-GFP cells, TAM, CAF, and EC accounted for the vast majority of the cells in tumor (Supplementary Fig. 4). To detect the cellular uptake of bLP in tumor, CAF, TAM, and EC were, respectively, labeled with specific antibodies for the measurements. In the captured images, CAF were denoted as α-smooth muscle actin (α-SMA)-positive (green signals), but CD31-negative (white signals) cells (α-SMA^+^/CD31^−^) (yellow arrows). Meanwhile, EC were depicted as CD31-positive cells with white signals (white arrows), and TAM were marked with specific antibody of F4/80 (green signals). In contrast to the D-Lipo group, the red fluorescence signals of D-bLP and DM-bLP were largely co-located with the specific signals of CAF, EC, and TAM (Fig. 3e–f; Supplementary Figs. 5, 6), suggesting the extensive uptake by these cells.

Next, we measured the accessibility of various nanosystems to cancer cells in tumor. The 4T1 cells with stable expression of green fluorescence protein (4T1-GFP) was used to develop the tumor models. The red signals of D-bLP and DM-bLP (red arrows) were mainly located nearby, but inaccessible to the 4T1-GFP cell regions in tumor (green signals) (Fig. 3g; Supplementary Fig. 7). Despite the significantly enhanced tumor accumulation and penetration of bLP versus counterpart D-Lipo, bLP were largely internalized by stromal cells and poorly accessed to cancer cells in tumor, which came into a great limit to exerting the therapeutic effects. On the other side, the flexible permeation of D-bLP in tumor and their extensive uptake by stromal cells provided an opportunity to remodel the TSM barriers for antitumor therapy.

### TSM remodeling by D-bLP-mediated photothermal effects

**Fig. 4:**
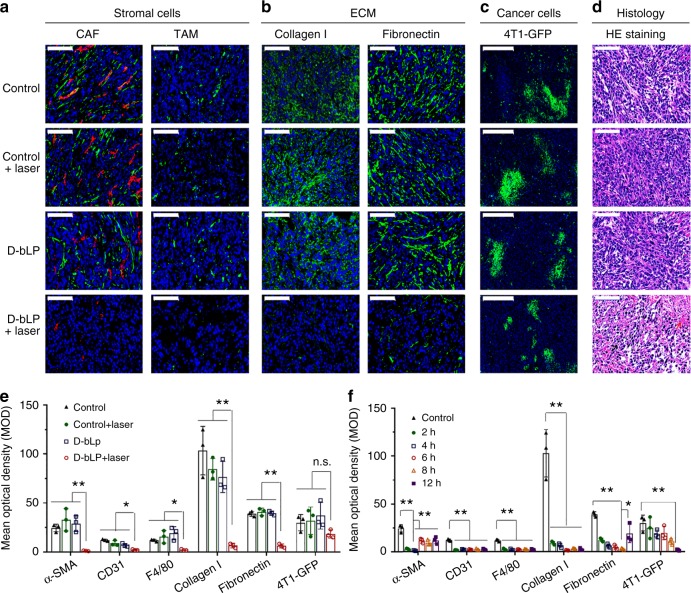
Efficient remodeling of TSM by D-bLP-mediated photothermal effects. At 12 -h post injection of D-bLP, one tumor was exposed to 808 -nm laser at 2.5 W cm^−2^ for 5 min, and the other one was not irradiated. These tumor tissues were collected at 4 h after laser irradiation for further measurements (**a**–**e**). **a** Variation of CAF and TAM populations in tumor with and without laser irradiation, scale bar, 100 µm. CAF were characterized by α-SMA^+^/CD31^−^ cells, which was denoted as cells with green signals, excluding red signals; TAM were presented as cells with green signals (F4/80). **b** Expression of collagen I and fibronectin in tumor with and without laser irradiation (green signals), scale bar, 100 µm. **c** Proportion of 4T1-GFP cancer cells (green signals) in tumor with and without laser irradiation, scale bar, 500 µm. **d** Histological examinations of tumors with and without laser irradiation by hematoxylin and eosin (H&E) staining, scale bar, 100 µm. Black arrow, karyopyknosis; red arrow, hemorrhage. **e** The quantified results of CAF, TAM, collagen I, fibronectin, and 4T1-GFP cancer cells in tumor with and without laser irradiation (*n* = 3). **f** Quantified variations of CAF, TAM, collagen I, fibronectin, and 4T1-GFP cancer cells in the D-bLP + laser-treated group at different time points after laser irradiation (*n* = 3). The data are means ± SD, *p < 0.05, **p < 0.01, n.s. not significant (ANOVA and two-tailed Student’s *t* test)

The stromal cells (e.g., CAF and TAM) and multiple ECM components (e.g., collagen, fibronectin) are the major ingredients of TSM^15,44^. The hyperthermia induced by laser irradiation could cause massive cell damages and significant denaturation of multiple ECM components^23,47–50^. To elucidate the impact of D-bLP-mediated photothermia on TSM barriers, we developed a two-tumor model with one tumor mass in the left side and another one in the right side in a same mouse. At 12- h post injection of D-bLP, one tumor mass was irradiated with an 808 -nm laser, and the other one was not irradiated. The tumor model without D-bLP injection was performed in the same procedure. At 4.0 -h post irradiation, the signals of CAF (green signals of α-SMA, excluding red signals of CD31) and TAM (green signals of F4/80) could be readily observed in control, control + laser and D-bLP-treated tumors (Fig. 4a). However, in D-bLP + laser-treated tumor, these signals were remarkably weakened, and the mean optical density (MOD) value was drastically decreased by 95.4% for α-SMA, 81.9% for CD31, and 81.3% for F4/80 in comparison with the untreated control tumors (Fig. 4a, e). Likewise, the ECM components of collagen I and fibronectin were severely downregulated in the D-bLP + laser group, but rarely impacted in other three groups (Fig. 4b). The expression of collagen I and fibronectin in D-bLP + laser tumor was notably reduced by 93.7% and 84.3% versus untreated control tumors (Fig. 4e).

With the prolongation of time, the expression of CD31, F4/80, and collagen I in D-bLP-treated tumor was rarely changed and remained at a low level within 12 h (Fig. 4f; Supplementary Figs. 8, 9, 10). However, the signals of α-SMA and fibronectin in D-bLP + laser-treated tumor changed from declining to rising with time, and were, respectively, recovered to about 50% at 6.0 -h and 12.0 -h post irradiation (Fig. 4f; Supplementary Figs. 8, 11). Compared with the control tumor, the MOD values of 4T1-GFP signals in D-bLP + laser group were not significantly reduced at 4 -h post irradiation, but drastically decreased to 6.0% at 12 h after irradiation (Fig. 4c–f; Supplementary Fig. 12). Histological examinations of these tumor tissues showed the cells in D-bLP + laser tumor became loosely packed with extensive karyopyknosis, hemorrhage (red arrow), and necrosis when compared with other control tumors (Fig. 4d; Supplementary Fig. 13). Thus, the D-bLP-mediated photothermia produced a remarkable reduction of CAF and TAM proportions, and caused drastic denaturation of collagen I and fibronectin in tumor. The massive disruption of TSM barriers could be ascribed to the flexible permeation of D-bLP in tumor, their extensive internalization by various stromal cells and efficient photothermal effects.

### Impact on tumor accumulation and penetration of M-bLP

**Fig. 5:**
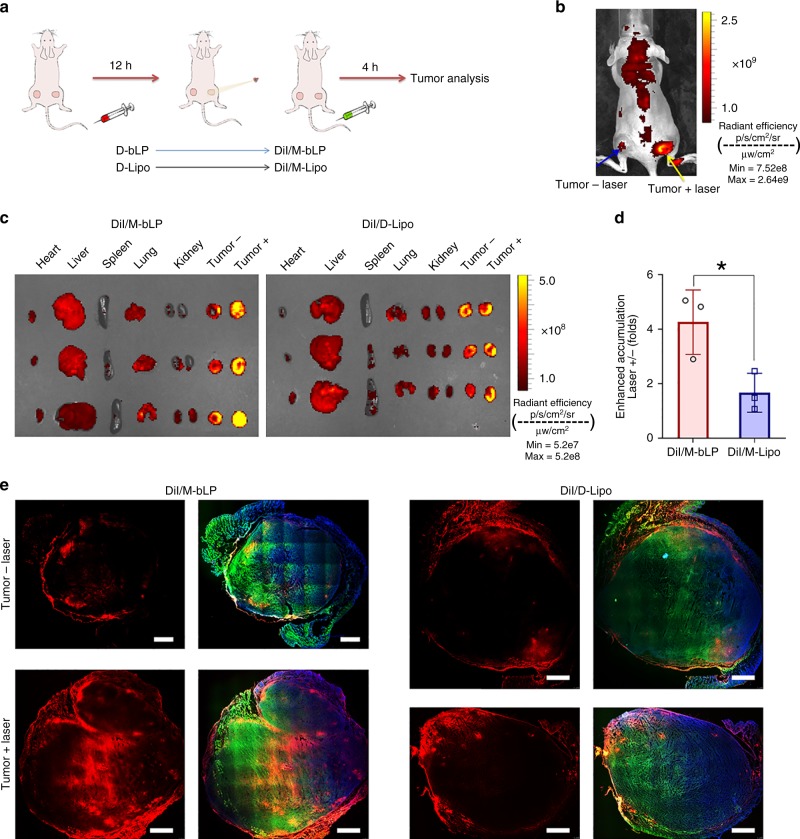
Impact of D-bLP-mediated TSM remodeling on tumor accumulation and penetration of second DiI/M-bLP. **a** Schematic illustration of the treatments in the two-tumor model, in which one tumor was irradiated at 12 h after injection D-bLP or D-Lipo, and the other one was not irradiated as a control, then followed with the second injection of DiI/M-bLP or DiI/M-Lipo via tail vein. The measurements were performed at 4 -h post injection. **b** Distribution of second DiI/M-bLP in a two-tumor model after D-bLP-mediated TSM remodeling. **c** The ex vivo imaging of DiI/M-bLP and DiI/M-Lipo in major organs from a two-tumor model after TSM remodeling (*n* = 3). **d** The enhanced accumulation of second DiI/M-bLP or DiI/M-Lipo in tumor with laser irradiation (*n* = 3). The data are means ± SD, *p < 0.05 (two-tailed Student’s *t* test). **e** Penetration of DiI/M-bLP and DiI/M-Lipo in tumor mass with and without laser irradiation, wherein the actin and nuclei were, respectively, stained with phalloidin-FITC and DAPI for visualization under LCSM, scale bar, 1.0 mm

To understand the impact of D-bLP-mediated TSM remodeling on tumor accumulation and penetration of second nanoparticles, M-bLP was labeled with DiIC18(3) (DiI) (DiI/M-bLP) for the measurements, which would not interfere with the fluorescence signals of D-bLP. In the two-tumor model at 12 -h post injection of D-bLP or D-Lipo, one tumor mass was irradiated at 808 -nm laser for 5 min, and the other one was not irradiated as a control, and then, respectively, injected with second DiI/M-bLP or DiI-labeled M-Lipo (DiI/M-Lipo) (Fig. 5a; Supplementary Fig. 14). The ex vivo imaging results indicated that the second DiI/M-bLP had better to be injected immediately after the laser irradiation (0 h) (Supplementary Fig. 15). In the D-bLP-treated group, the fluorescence signals of second DiI/M-bLP were largely detected with strong intensity in laser-irradiated tumor, which was 4.27-fold higher than that in untreated tumor (Fig. 5a–d). However, in D-Lipo-treated group, the fluorescence intensity of second DiI/M-Lipo was only slightly enhanced in laser-irradiated tumor (Fig. 5c, d). Thereby, D-bLP-mediated TSM remodeling caused notable improvement of second DiI/M-bLP accumulation in tumor, which was superior to the counterpart liposomal formulations.

Then, the impact of D-bLP-mediated TSM remodeling on tumor penetration of second DiI/M-bLP was measured using LCSM. In Fig. 5e, the red fluorescence signals of DiI/M-bLP in laser-treated tumor mass were widely visualized in the whole tumor mass with stronger intensity versus the untreated tumor. However, in D-Lipo-treated groups, the signals of second DiI/M-Lipo were mainly restricted in the exterior regions of tumor mass with mild penetration in laser-irradiated tumor (Fig. 5e; Supplementary Fig. 16). These results confirmed the merit of D-bLP-mediated TSM remodeling on enhancing tumor penetration of second-wave M-bLP, which was more efficient than the counterpart liposomal formulations. The stromal cells and the ECM components constitute of the crucial TSM barriers that hamper nanoparticles permeation in tumor mass^51,52^. The CAF and TAM in tumor were remarkably eliminated by D-bLP-mediated photothermia, but poorly impacted by D-Lipo-mediated photothermal treatments (Supplementary Figs. 17, 18). The massive disruption of TSM barriers by D-bLP-mediated photothermia would clear up the intratumoral delivery obstacles and facilitate the permeation of second DiI/M-bLP in tumor mass. In addition, the small particle size and bioinspired properties of second M-bLP were necessary for the enhanced accumulation and permeation in tumor.

### Impact on M-bLP extravasation from tumor vasculature

**Fig. 6:**
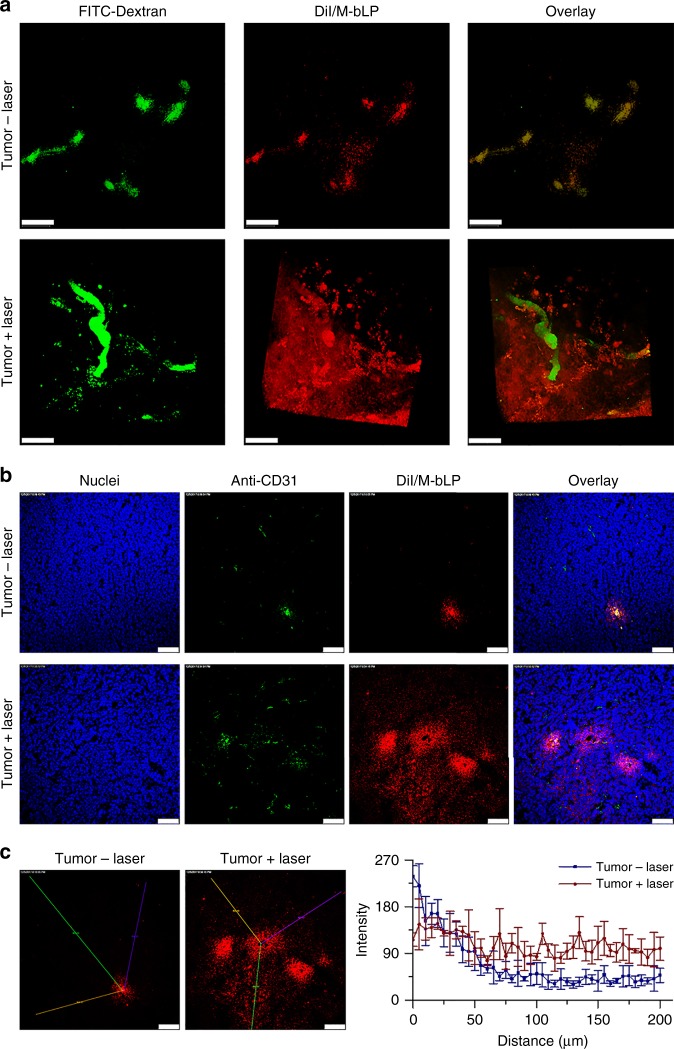
Impact of D-bLP-mediated TSM remodeling on extravasation of second DiI/M-bLP from tumor vasculatures. **a** Extravasation of DiI/M-bLP from tumor vessels of untreated and laser-irradiated tumors, scale bar, 100 µm. **b** Diffusion of DiI/M-bLP from tumor vessels into the distal matrix in tumor with and without laser irradiation, scale bar, 75 µm. **c** Quantified diffusion of DiI/M-bLP from tumor vessels to the distal matrix by Image J software. The data are means ± SD, *n* = 3

The nanoparticles extravasation from tumor vessels is an essential prerequisite for their accessibility to cancer cells in tumor^1,2,6^. To elucidate the effect of TSM remodeling on nanoparticles extravasation from tumor vasculature, the tumor models were treated as described above, and the blood vessels were labeled with fluorescein isothiocyante (FITC)–dextran for visualizing under LCSM. In untreated tumor, the fluorescence signals of DiI/M-bLP were mainly trapped within the vessel lumen or in the matrix close to the tumor vessels (Fig. 6a). Surprisingly in laser-irradiated tumor, the red signals from DiI/M-bLP could be extensively detected in distal regions from tumor vasculatures. The extravasation of DiI/M-bLP from tumor vessels was also validated in the transversal profiles (Fig. 6b). The calculated results indicated that DiI/M-bLP could diffuse only 50 µm away from the tumor vessels in untreated control tumor, but efficiently permeate through over 200 µm from tumor vessels with high fluorescence intensity in laser-irradiated tumor (Fig. 6c). These results confirmed the validity of D-bLP-mediated TSM-remolding on enhancing second M-bLP extravasation from tumor vasculature into the distal matrix, thereby holding great potential to facilitating their accessibility to cancer cells.

### Impact on M-bLP accessibility to cancer cells

**Fig. 7:**
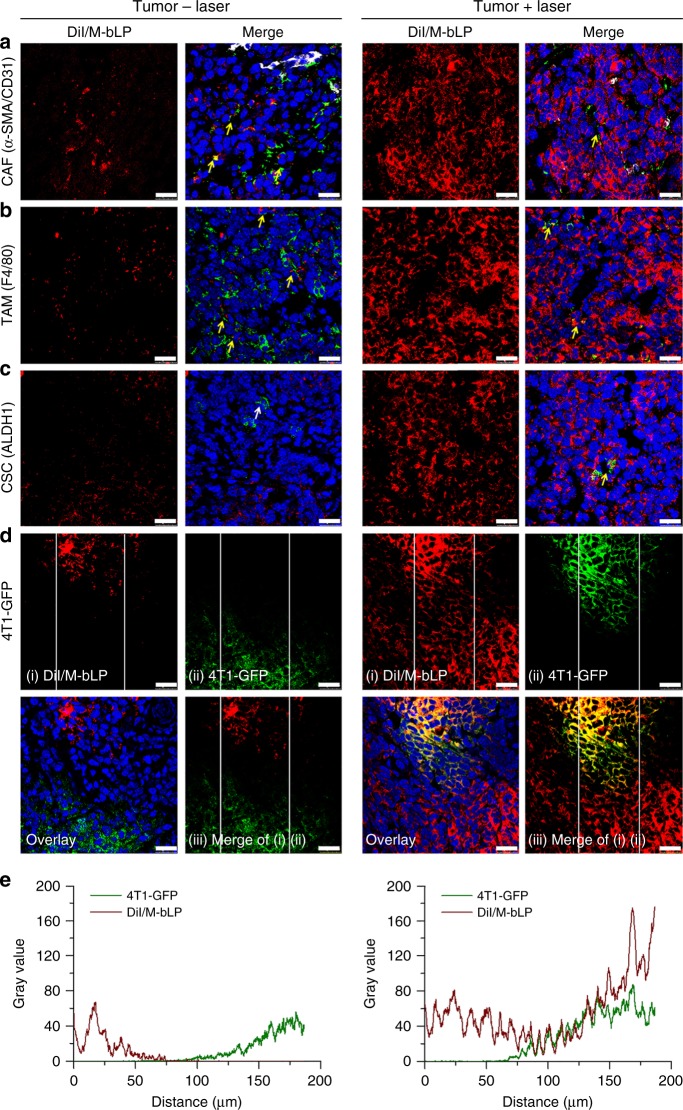
Impact of D-bLP-mediated TSM remodeling on second DiI/M-bLP accessibility to cancer cells in tumor. The uptake of DiI/M-bLP by various cells in tumor with and without laser irradiation was visualized under LCSM, scale bar, 25 µm. **a** CAF, which was denoted as α-SMA-positive (green signals) but CD31-negative cells (white signals) (α-SMA^+^/CD31^−^). **b** TAM (green signals). **c** ALDH^high^ cancer cells (green signals). **d** 4T1-GFP cancer cells (green signals). **e** Accessibility of second DiI/M-bLP to cancer cells regions in control tumor and laser-irradiated tumor. The images between two white lines in panel (iii) of Fig. **d** was analyzed by the Image J software

To determine the impact of D-bLP-mediated TSM remodeling on M-bLP accessibility to cancer cells, the 4T1- and 4T1-GFP-induced two-tumor model were used for the measurements. The CAF and TAM in tumors were specifically labeled for visualization under LCSM (Fig. 7a, b). In the D-bLP treated group, the signals of DiI/M-bLP were largely co-localized with that of CAF and TAM in untreated control tumor (yellow arrows), but barely merged with them in laser-irradiated tumor (Fig. 7). By contrast, in D-Lipo-treated groups, the second DiI/M-Lipo was obviously co-localized with these stromal cells in laser-irradiated and un-irradiated tumors (Supplementary Figs. 17–19). The reduced internalization of second DiI/M-bLP by stromal cells versus counterpart DiI/M-Lipo could be ascribed to the remarkable elimination of TAM and CAF by D-bLP-mediated photothermia, which would be beneficial to promoting their accessibility to cancer cell fractions in tumor.

Importantly, in 4T1-GFP-induced tumor model, the co-localization of second DiI/M-bLP in 4T1-GFP cells regions was evidently observed in laser-irradiated tumor, but merely visualized in untreated control tumor (Fig. 7d). In untreated control tumor mass, the red signals of DiI/M-bLP were halted at about 20–50 µm away from the 4T1-GFP cell regions in tumor and rarely accessed the 4T1-GFP cancer cells (Fig. 7d, e). However, in tumors with D-bLP-mediated photothermal treatments, the red signals of DiI/M-bLP efficiently reached the 4T1-GFP cells regions and maintain at a high intensity (Fig. 7d, e). The quantified analysis showed that the mean fluorescence intensity of DiI/M-bLP in 4T1-GFP cancer cell regions was 27.0-fold higher than that in untreated control tumor (*p* < 0.01) (Supplementary Fig. 20). In addition, cancer stem-like cells (CSCs), the root of cancer relapse and metastasis^53,54^, were characterized by aldehyde dehydrogenase-positive (ALDH^+^) cells. In laser-irradiated tumor mass, DiI/M-bLP could be internalized by the ALDH^+^ CSCs (Fig. 7c). In the counterpart D-Lipo group, the second DiI/M-Lipo was unable to reach the 4T1-GFP cells regions in tumor (Supplementary Fig. 19). Therefore, the D-bLP-mediated TSM remodeling could enhance the accessibility of second M-bLP to cancer cells or even CSCs in tumor, which was superior to the counterpart liposomal formulations.

### In vitro therapeutic efficacy on metastatic 4T1 cells

**Fig. 8:**
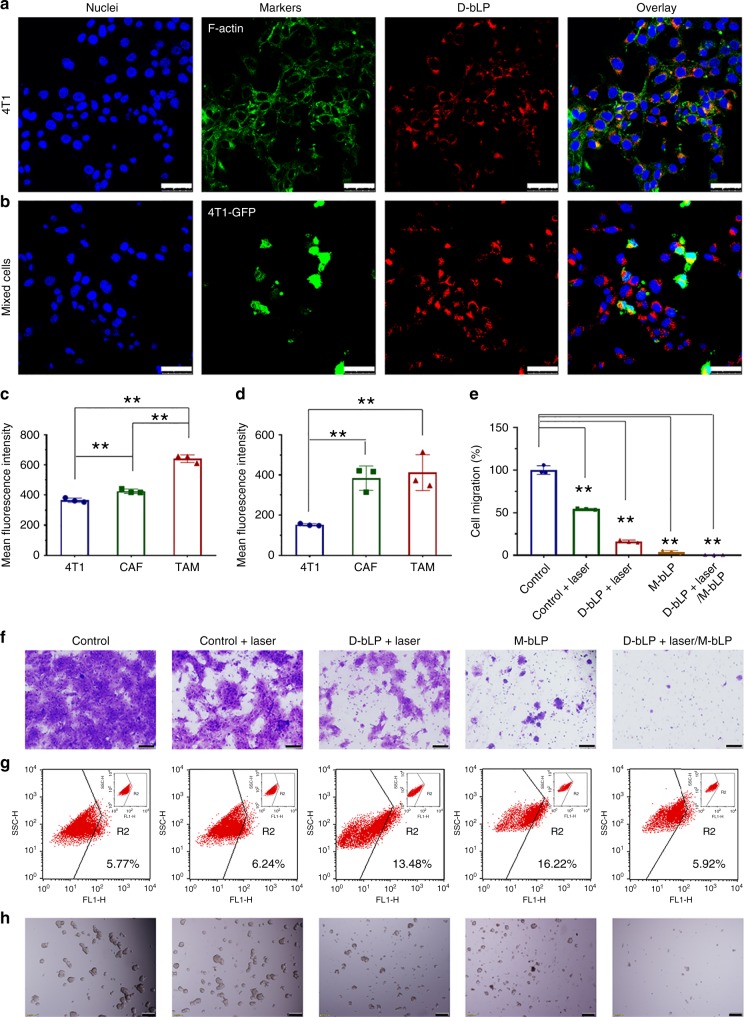
The in vitro therapeutic effects on the viability, migration, and stemness of 4T1 cancer cells. Cells were, respectively, treated with laser, D-bLP + laser (10 µg·mL^−1^ of DiR), M-bLP (1.0 µg·mL^−1^ of mertansine), and their combination of D-bLP + laser/M-bLP. **a** Cellular uptake of D-bLP by 4T1 cells under LCSM, scale bar, 50 µm. **b** Cellular uptake of D-bLP by 4T1-GFP cancer cells in the mixed-cell model, scale bar, 50 µm. **c** Quantified cellular uptake by each cell type in the single-cell model (*n* = 3). **d** Quantified cellular uptake by each cell type in thre mixed-cell model (*n* = 3). **e** Quantified anti-migration effects of these treatments (*n* = 3). **f** Migration of 4T1 cells across the transwell membrane from each treatment, scale bar, 100 µm. **g** Proportion of ALDH^high^ CSCs in 4T1 cells after these treatments. **h** Tumorsphere-forming ability of 4T1 cells after these treatments, scale bar, 200 µm. The data are means ± SD, **p < 0.01 (ANOVA and two-tailed Student’s *t* test)

The therapeutic effects of D-bLP, M-bLP, and their synergistic combination were evaluated in metastatic 4T1 breast cancer cells. The uptake of D-bLP by cancer cells (4T1), TAM (RAW 264.7), and CAF were, respectively, determined in the single-cell phenotype model and mixed-cell phenotype model. CAF were isolated from 4T1-induced tumor mass and characterized by α-SMA^+^/CD31^−^ cells (Supplementary Fig. 21). In the single-cell phenotype model, D-bLP was extensively taken up by 4T1, RAW 264.7, and CAF with strong intensity (Fig. 8a; Supplementary Fig. 22a), and the mean fluorescence intensity in TAM and CAF was higher than that in 4T1 cells (*p* < 0.01) (Fig. 8c). Then, these cells were mixed at 1:1:1 and co-incubated overnight to develop the mixed-cell phenotype model. The ratio of 4T1 cancer cells, TAM, and CAF were analyzed by flow cytometer to monitor the measurements (Supplementary Fig. 23). D-bLP could be efficiently internalized by each cell phenotype (Supplementary Fig. 22b), and the mean fluorescence intensity in CAF and TAM was obviously higher than that in 4T1 cancer cells (Fig. 8b, d) (*p* < 0.01). Thus, D-bLP could be preferentially taken by TAM and CAF versus 4T1 cancer cells in these two models.

The bLP can be efficiently internalized by a variety of cancer cells from murine or human^31–33^. In the formulation of D-bLP and M-bLP, ApoA-1 mimic peptide was an essential ingredient. Notably, ApoA1 was a major protein in natural HDL, which could bind to the scavenger receptor class B type I (SR-BI) to promote their internalization by various cells^40^. Moreover, the amino acid sequence of SR-BI shares 30% homology with that of CD36^40^. SR-BI was highly expressed in TAM and CAF versus 4T1 cancer cells, and CD36 was exceedingly expressed in TAM over CAF and 4T1 cells (*p* < 0.01) (Supplementary Fig. 24). When cells were pretreated with specific antibodies against CD36, SR-BI or their combinations, the uptake of D-bLP was barely changed in 4T1 cells (*p* > 0.05), but greatly reduced in TAM (*p* < 0.01) (Supplementary Fig. 25). In CAF, the uptake of D-bLP was rarely decreased in anti-CD36-treated group (*p* > 0.05), but significantly reduced in cells treated with anti-SR-BI or their combinations. Therefore, the preferential uptake of D-bLP in TAM or CAF over 4T1 cancer cells would be associated with the highly expressed SR-BI and its synergistic effects with CD36.

Then, the inhibitory effects of D-bLP + laser, M-bLP, and their combinations on the viability and migration activities of 4T1 cells were investigated. The viability of 4T1 cells was mildly suppressed by D-bLP + laser (10 µg·mL^−1^ of DiR) or M-bLP (1.0 µg·mL^−1^ of mertansine) treatments, and significantly reduced by 61.7% in D-bLP + laser/M-bLP (Supplementary Fig. 26). Then, the migration of these residual cells were measured using a transwell-mediated assay (Fig. 8e, f). Compared with the negative group, the migration of 4T1 cells was obviously reduced by 45.8% in laser alone, 84.0% in D-bLP + laser and 96.4% in M-bLP-treated group, and almost negligible in the D-bLP + laser/M-bLP group. Even when cells were incubated at the concentration that had no inhibition on cell viability (Supplementary Fig. 27), the migration of 4T1 cells was efficiently inhibited by 93.1% in the D-bLP + laser/M-bLP group, which was more effective than single treatment of D-bLP + laser (51.0% at 0.1 µg·mL^−1^ of DiR) or M-bLP (60.7% at 0.01 µg·mL^−1^ of mertansine) (Supplementary Figs. 28, 29). Thereby, the D-bLP + laser/M-bLP treatment presented a remarkable suppression on cell migration.

CSCs have been proposed to account for the tumor metastasis and relapse after therapy^55,56^. The stemness of residual 4T1 cells was characterized by ALDH-positive cells. The flow-cytometer assays showed that the proportion of ALDH^high^ cells was 5.77% in negative control, but unexpectedly enriched to 13.48% in the D-bLP + laser group (10 µg·mL^−1^ of DiR) and 16.22% in the M-bLP group (1.0 µg·mL^−1^ of mertansine). Interestingly, in the D-bLP + laser/M-bLP group, the proportion of ALDH^high^ cells was reverted to 5.92%, which was comparable with that in negative control, but less than that in single treatment of D-bLP + laser or M-bLP (Fig. 8g). Moreover, the self-renewal capacity of these residual cells was characterized by sphere-forming assays. Different from other groups, only small fragmentary spheres or single cells were visualized in the D-bLP + laser/M-bLP group (Fig. 8h). Thus, the D-bLP + laser/M-bLP treatment could abate the enrichment of CSCs fractions and inhibit the self-renewal capacity, holding great premise to suppress tumor relapse and metastasis.

### In vivo therapeutic effects on tumor metastasis and relapse

**Fig. 9:**
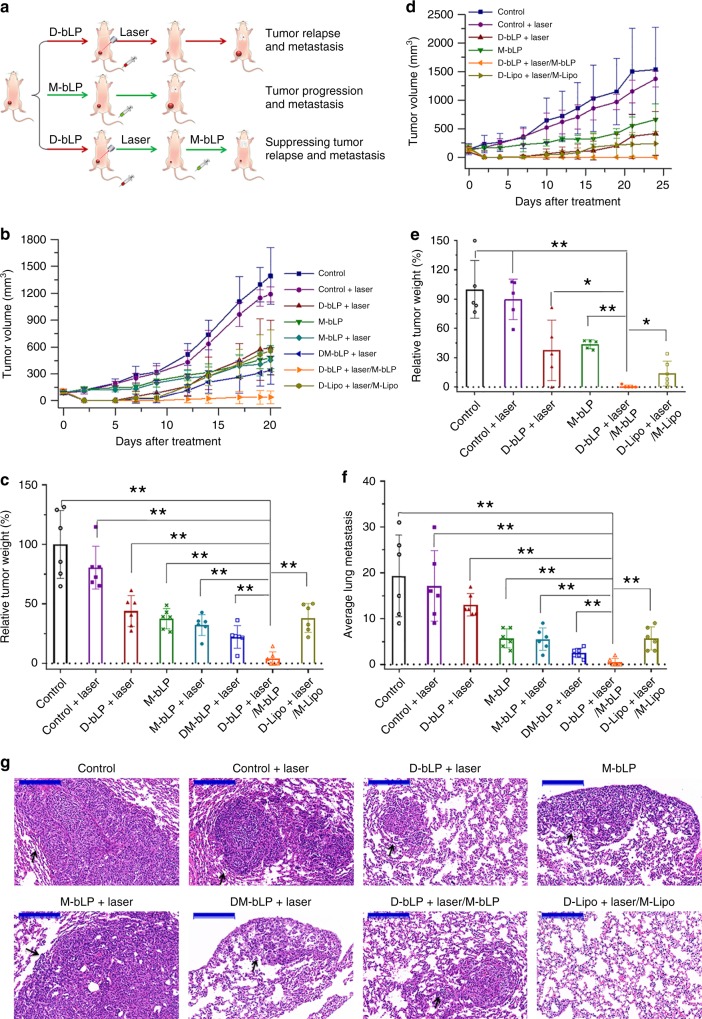
The in vivo therapeutic efficacy of TECA strategy on tumor relapse and metastasis in the breast cancer model. **a** Schematic illustration of the treatments. **b** The tumor growth profiles from each group in 4T1-induced tumor model (*n* = 6). **c** The relative tumor weight from each group in the 4T1-induced tumor model (*n* = 6). **d** The tumor growth profiles from each group in the MCF-7-induced tumor model (*n* = 5). **e** The relative tumor weight from each group in the MCF-7-induced tumor model (*n* = 5). **f** The average number of lung metastatic nodules from each group in the 4T1-induced metastatic model (*n* = 6). **g** Histological examination of lung tissues from each group in the 4T1-induced metastatic model. Black arrows, metastatic lesion in lungs, scale bar, 200 µm. The data are means ± SD, *p < 0.05, **p < 0.01 (ANOVA and two-tailed Student’s *t* test)

Finally, the therapeutic effects of bLP-mediated TECA strategy on tumor relapse and metastasis were measured in two orthotopic breast cancer models (Fig. 9a). In 4T1-induced metastatic tumor model, mice were, respectively, treated with 12 groups (*n* = 6). The surface temperature at tumor sites from each NIR laser-irradiated group were monitored (Supplementary Fig. 30). The treatments of D-bLP + laser and DM-bLP + laser showed a considerable shrinkage of the tumor size within first 5 days, but unfortunately regrew thereafter (Fig. 9b). By contrast, the M-bLP and M-bLP + laser treatments produced moderate inhibition of tumor growth. Particularly in the D-bLP + laser/M-bLP group, tumors were obviously shrunk at first 9 days and completely abated except 3 of 6 mice, leading to a remarkable inhibition on tumor growth and effective prevention of tumor relapse (Fig. 9b; Supplementary Fig. 31 and Supplementary Table 2). Moreover, the D-bLP + laser/M-bLP treatment was superior to the counterpart D-Lipo + laser/M-Lipo and other liposomal formulations treated groups (Fig. 9b, c; Supplementary Fig. 32). We also weighed the tumor mass from each group and confirmed the efficient antitumor effects of D-bLP + laser/M-bLP treatment, which showed a 95.9% inhibition of tumor growth (Fig. 9c). Likewise, in the MCF-7-induced human tumor model (Supplementary Fig. 33) (*n* = 5), the D-bLP + laser/M-bLP treatment resulted in complete tumor ablation in four of five mice and produced a 99.4% inhibition of tumor growth, which was far more effective than that of D-Lipo + laser/M-Lipo and other groups (Fig. 9d, e; Supplementary Fig. 34 and Supplementary Table 2). In addition, the reductions of body weights from each treatment were rarely detected in these two-tumor models (Supplementary Figs. 35, 36). Therefore, the D-bLP + laser/M-bLP treatment produced efficient suppression of tumor growth and relapse.

Lungs are one of the most frequent organs of breast cancer metastasis^3,57^. At the end point of treatment in the 4T1-indued tumor model, the lung metastatic nodules were detected in only two of six mice in the D-bLP + laser/M-bLP group, but evidently observed in all mice of other groups (Fig. 9f, g; Supplementary Fig. 32d and Supplementary Table 3). Surprisingly, in the D-bLP + laser/M-bLP group, the average metastatic nodules in the lung was only 0.5 ± 0.8, resulting in a 97.4% inhibition of lung metastasis in comparison with the negative control. Moreover, the average lung metastatic nodules in the D-bLP + laser/M-bLP group was only 3.8% of the D-bLP + laser group, 8.8% of M-bLP, 9.1% of M-bLP + laser, 8.8% of the D-Lipo + laser/M-Lipo counterpart, and 20% of the combined DM-bLP + laser group, which effectively validated the outstanding antimetastatic efficacy of D-bLP + laser/M-bLP treatment (Fig. 9f). In addition, the histological examinations of these lung tissues confirmed the prominent therapeutic effects of D-bLP + laser/M-bLP treatment on lung metastasis (Fig. 9g; Supplementary Figs. 37, 38). Therefore, the bLP-mediated TECA strategy plays an essential role for the efficient suppression of tumor relapse and metastasis.

## Discussion

Tumor relapse and metastasis are the primary causes of poor survival rates in patients with advanced cancer^58^. Cancer cells are the initial insult of malignant tumors, but they are co-evolved with a variety of stromal cells to promote tumor progression and metastasis^13,44,59^. Of note, multiple stromal cells (e.g., CAF and TAM) and the dense network of ECM constitute of the TSM barriers hampering drug accessibility to cancer cells in tumor, thereby severely compromising their therapeutic efffects^13–15^. Although nanosized particles are promising for tumor targeted drug delivery, they are often restricted around the tumor vessels and poorly accessed to cancer cells in tumor^9,17,18^. In this work, although the small-sized bioinspired lipoproteins of D-bLP, DM-bLP, and DiI/M-bLP can considerably accumulate in tumor and penetrate the tumor mass (Figs. 3, 5; Supplementary Fig. 16), they are largely hijacked by stromal cells and unable to reach the cancer cells in tumor (Figs. 3, 7; Supplementary Figs. 5–7). Herein, the bLP-mediated TECA strategy efficiently disrupt the TSM barriers and exceedingly promote the accessibility of second M-bLP to cancer cells in tumor, and ultimately produced prominent therapeutic outcomes on tumor relapse and metastasis, which was superior to the counterpart liposomal formulations.

The photothermal effects present unique advantages of high specificity and precise spatial-temporal selectivity in cancer treatments, making it a powerful tool for TSM remodeling^20,23,48,50^. In this study, D-bLP showed efficient tumor accumulation, deep tumor penetration, and extensive internalization by diverse stromal cells at tumor sites, providing a substantial opportunity to TSM remodeling. Upon their exposure to laser irradiation, the CAF and TAM in tumor were remarkably eradicated, and the expression of collagen I and fibronectin was drastically lowered, resulting in the massive disruption of TSM barriers (Fig. 4). By contrast, the counterpart liposomal D-Lipo exhibited poor permeation in tumor and produced limited effectiveness on TSM remodeling upon their exposure to laser irradiation (Supplementary Figs. 17, 18). These results confirmed the superiority of D-bLP over D-Lipo in TSM remodeling, which was mainly ascribed to the higher tumor accumulation, deeper tumor penetration, and extensive internalization by stromal cells of D-bLP versus D-Lipo (Fig. 3; Supplementary Figs. 5, 6).

Recent evidence reveals that the remodeling of stromal cells or ECM would be feasible to improve nanoparticles accumulation and penetration in tumor^51,52^. The massive disruption of TSM barriers by D-bLP-mediated photothermal effects would facilitate the tumor accumulation and cancer cell accessibility of second M-bLP, which was validated in the metastatic 4T1 tumor model (Figs. 5–7). The D-bLP-mediated TSM remodeling caused a 4.27-fold enhancement of DiI/M-bLP accumulation in tumor and deeper penetration in tumor mass when comparing to the un-irradiated tumor. Moreover, upon the D-bLP-mediated TSM remodeling, the second delivered DiI/M-bLP would exceedingly permeate from tumor vessel into the distal matrix and efficiently reach the 4T1-GFP cell regions in tumor, ultimately producing a 27.0-fold increase of second DiI/M-bLP accessibility in 4T1-GFP cell regions (Supplementary Fig. 20). However, in the liposomal D-Lipo-mediated TSM remodeling, the tumor accumulation and penetration of second DiI/M-Lipo were mildly enhanced. and they are ineffective to access the 4T1-GFP cell regions in tumor (Fig. 5e; Supplementary Fig. 19). These results effectively verified the superiority of bLP-mediated TECA strategy on enhancing second nanoparticles accessibility to the cancer cells in tumor, which was primarily owing to the effective D-bLP-meditated TSM remodeling, as well as the small particle size and bioinspired properties of M-bLP.

The therapeutic benefits of bLP-mediated TECA strategy were evaluated in two breast tumor models. In murine 4T1-induced tumor model, the D-bLP + laser treatment following with continuous M-bLP (D-bLP + laser/M-bLP treatment) produced 95.9% inhibition on tumor growth and 97.4% suppression of lung metastasis. Moreover, tumor relapse was observed in 3/6 mice, and the visualized lung metastases were merely detected in 2/6 animals in the D-bLP + laser/M-bLP group. Likewise, in the MCF-7-induced tumor model, the D-bLP + laser/M-bLP treatment successfully prevented tumor relapse in 4/5 mice. These results verified the prominent therapeutic efficacy of D-bLP + laser/M-bLP treatments on suppressing tumor relapse and distant metastasis, which was more efficient than the counterpart D-Lipo + laser/M-Lipo treatments. In addition to the smaller particle size and bioinspired properties of bLP versus counterpart liposomal formulations, the D-bLP-mediated TECA strategy would certainly account for the notable therapeutic outcomes on tumor relapse and metastasis (Fig. 9).

However, this study is restricted to the treatment of primary tumor, and would be invalid to treat metastatic cells or micrometastases in distant organs. The notable inhibition of lung metastasis in the 4T1 tumor model could be presumably ascribed to the reduction of primary tumor rather than direct eradiation of metastasis. Of note, tumor cells dissemination is an early event in breast cancer metastasis, and eradicating the disseminated cancer cells in primary tumor will be a feasible approach to prevent cancer metastasis^55,56^. In this study, the bLP-mediated TECA strategy remarkably improve second nanoparticles accessibility to cancer cells or even CSCs in tumor, providing an encouraging approach to treat tumor relapse and metastasis. Distinctly, further studies to ameliorate TECA strategies by exploring sophisticated nanosystems or alternative TSM-remodeling modalities will be preferred for cancer therapy.

## Methods

### Materials

DMPC and DOPE were purchased from Shanghai Advanced Vehicle Technology Pharmaceutical Ltd (Shanghai, China). The ApoA1-mimetic peptide (PK-22) was synthesized by GL Biochem (Shanghai) Ltd (Shanghai, China). Mertansine was supplied by BrightGene BioMedical Technology CO. Ltd (Jiangsu, China). DiR was supported by Amyjet Scientifc Inc. (Wuhan, China), and DiI was obtained from Beyotime Institute of Biotechnology (Jiangsu, China). Natural mature HDL from human plasma (lyophilized powder, L1567) was supplied by Sigma-Aldrich (MO, USA).

The murine 4T1 cells, RAW 264.7 cells, and human MCF-7 cells were provided from Cell Bank of Shanghai, Chinese Academy of Sciences (CAS, Shanghai, China). The 4T1-GFP cells were obtained from Keyuandi Biotech Co. Ltd (Shanghai, China). The 4T1, 4T1-GFP, and MCF-7 cells were kept in RPMI-1640 media with 10% FBS (Gibco), and cultured at 37 °C and 5% CO_2_ in a humidified incubator for further measurements. RAW 264.7 were incubated with Dulbecco’s Modified Eagle Medium (DMEM) with 10% FBS, and cultured as described above. Female nude mice (18–22 g) were provided by Shanghai Experimental Animal Center, CAS (Shanghai, China) and used to induce the breast cancer tumor model. The animal experiments were performed according to the protocols approved by the Institutional Animal Care and Use Committee (IACUC) of Shanghai Institute of Materia Medica, CAS.

### Preparation and characterization of D-bLP and M-bLP

For preparing D-bLP, DMPC (6 mg), DOPE (4 mg), and DiR (0.5 mg) were dissolved in chloroform/methanol (1:1, v/v) in a round flask. The mixed solution was evaporated under vacuum at 30 °C to form a uniform lipid film. Then, the film was dispersed with 1.0 mL of distilled water and sonicated in ice bath for 2.5 min to prepare the liposomal formulations (denoted as D-Lipo). Thereafter, 2 mg of PK-22 was mixed with D-Lipo, and performed three heating–cooling circles between 50 °C and 6 °C to obtain the D-bLP. In a similar procedure, M-bLP, DM-bLP, and DiI/M-bLP as well as the counterpart liposomal formulations were fabricated for further measurements.

The morphologies of D-bLP, M-bLP, and natural mature HDL were visualized under FE-TEM (Tecnai G^2^ S-Twin, FEI) after negative staining with uranyl acetate solution, and then analyzed to calculate the mean diameters. The exact compositions in D-bLP, M-bLP, and natural mature HDL were, respectively, determined (Supplementary Table 1). The bLP system was filtered through a 0.22-μm membrane to remove the unentrapped lipophilic DiR or mertansine^33^, and then diluted with methanol for further measurements. The concentrations of DiR in D-bLP were measured by fluorescence analysis (Enspire, PerkinElmer, Singapore, Ex 748 nm; Em 780 nm). Meanwhile, the amount of mertansine in M-bLP was determined by a modified HPLC method^60^. The mobile phase was composed of acetonitrile with 0.1% trifluoroacetic acid (A) and water with 0.1% trifluoroacetic acid (B). The gradient elution program was modified as: 0–5 min, from 50% A to 90% A, 5–15 min, 90% A, 15–20 min, from 90% A to 50% A. The EE (%) values of DiR or mertansine were denoted as the amount of incorporated drug compared with the total drug amount in bLP or liposomal formulations. The DL capacity (%) was defined as the ratio between the amount of entrapped DiR or mertansine and the amount of all ingredients in bLP or liposomal formulations. The proportion of phospholipids in these formulations were determined by the phospholipid assay kit (Sigma). The concentration of protein in natural HDL was measured by a BCA protein assay (Beyotime, China), and that of peptides in bLP was quantified using an ultraviolet–visible spectrum analysis at a wavelength of 220 nm. The measurements were performed in triplicate.

To evaluate the stability of D-bLP and M-bLP as well as the counterpart liposomal formulations in physiological environments, 100 μL of these bLP were, respectively, diluted with 900 μL of PBS or whole FBS in triplicate, and further incubated for 24 h. At certain time intervals, samples were collected and filtered through a 0.22-μm membrane to separate the unentrapped DiR or mertansine. Then, samples were diluted with methanol and analyzed to calculate the percentage of remained drug in bLP or counterpart liposomes. The measurements were performed in triplicate.

The heat-generating ability of D-bLP upon laser irradiation was monitored using an infrared thermal camera (A150–15-M, Irtech Ltd). In brief, 100 μL of water, free DiR solution, D-Lipo, and D-bLP (200 µg·mL^−1^ of DiR) were, respectively, exposed to an 808-nm laser (MDL-N-10W, Changchun New Industries, China) at 2.0 W cm^–2^ for 5 min. The thermographic images and the temperature changes during laser irradiation were recorded using the infrared thermal camera. The measurements were performed in triplicate.

### In vivo tumor targeting in a metastatic breast cancer model

The in vivo tumor targeting capability of D-bLP, DM-bLP, D-Lipo, and free DiR were evaluated in a metastatic breast cancer tumor model, which was developed by injecting 4T1 cells to the fourth mammary pad of nude mice at one million cells per mouse. At day 10 after inoculation with the tumor volume around 100–150 mm, free DiR, D-bLP, DM-bLP, and D-Lipo were, respectively, injected to the tumor models at 5.0 mg·kg^−1^ of DiR via tail vein. At predetermined time points, mice were anaesthetized and the fluorescence signals were recorded using the in vivo imaging system (IVIS Spectrum, PerkinElmer). At 12.0 -h post injection, mice were killed, and the major organs including the heart, liver, spleen, lung, kidney, and tumor were carefully collected for imaging (*n* = 3). The mean fluorescent signals in each organ were analyzed by the accompanied software for quantification. Moreover, the intratumoral distribution of these nanosystems was monitored using the photoacoustic imaging system (Vevo LAZR, VisualSonic FUJIFILM). The photoacoustic signals of these nanosystems were presented as green signals, and the ultrasound signals of the tumors were collected as control.

Then, to elucidate their internalization by various cells in tumor, tumor tissues from each group were sectioned at 10 μm (CM1950, Leica, Germany) for visualization under LCSM (TCS-SP8 STED, Leica, Germany). To investigate their localization in CAF, samples were stained with the specific antibodies against a-SMA (Abcam, ab5694, 1:500) and CD31 (Abcam, ab7388, 1:250), and then incubated with the secondary antibodies of FITC-labeled IgG (H + L) (Beyotime, A0562, 1:400) or Cy3-labeled IgG (H + L) (Beyotime, A0507, 1:400), respectively. CAF were characterized by α-SMA-positive and CD31-negative cells (α-SMA^+^/CD31^−^), which was denoted as cells with green signals, excluding white signals. By contrast, cells with white signals were presented as EC of tumor vessels. Then, to evaluate their uptake by TAM, samples were stained with the specific antibody against F4/80 (Abcam, ab6640, 1:250), and then incubated with secondary antibody of Alexa Fluor 488-labeled IgG (H + L) (Yeasen, 33316ES60, 1:50) to outline the TAM (green signals) in tumor sections. By contrast, the nuclei were counterstained with 4′,6-diamidino-2-phenylindole (DAPI, Beyotime, C1002) for the observations. These measurements were repeated twice.

To determine their uptake by cancer cells, 4T1-GFP cells were used to develop the tumor model by injecting 4T1-GFP cells to the fourth mammary pad of nude mice at one million cells per mouse. D-bLP, DM-bLP, and D-Lipo were, respectively, injected to the tumor models at 5.0 mg·kg^−1^ of DiR via tail vein. At 12 h after injection, the tumor tissues were collected, sectioned at 10 μm, and then stained with DAPI for visualization under LCSM (TCS-SP8 STED, Leica, Germany). The cancer cells in tumor sections were denoted as clustered cells with green signals. The specific uptake of these nanosystems by cancer cells was presented as the co-location of red signals of various nanosystems and green signals of 4T1-GFP cells. These measurements were repeated twice.

### Remodeling TSM via D-bLP-mediated photothermal effects

To evaluate the impact of D-bLP-mediated photothermia on TSM, a two-tumor model was induced by injecting 4T1 cells to bilateral flanks of a mouse. When the tumor volume reached ~150 mm^3^, D-bLP was injected to the two-tumor model via tail vein. At 12 -h post injection, one tumor mass was exposed to 808 -nm laser at 2.5 W·cm^−2^ for 5 min, and the other one was untreated as a negative control. As control, the two-tumor model without D-bLP injection was performed in the same program. At 2.0 -h, 4.0 -h, 6.0 -h, 8.0 -h, and 12.0 -h post-laser irradiation, these tumors were collected, embedded in the paraffin, and sectioned for further assays. To evaluate the impact on CAF, sections were stained with specific antibodies of a-SMA (Servicebio, GB13036, 1:500) and CD31 (Servicebio, GB12063, 1:100), and then, respectively, incubated with FITC-labeled and Cy3-labeled secondary antibodies (Servicebio, GB22401, 1:200 and GB21404, 1:300). CAF were characterized by α-SMA^+^/CD31^−^ cells, which was denoted as cells with green signals, excluding red signals. Cells with red signals were denoted as the EC of tumor vessels. To evaluate their impact on TAM and ECM components, sections were, respectively, stained with the specific antibodies of F4/80 (Servicebio, GB13027, 1:100), collagen Ι (Abcam, ab21286, 1:200), and fibronectin (Servicebio, GB13091, 1:250), and then incubated with Alexa Fluor 488-labeled secondary antibodies (Servicebio, GB25303, 1:400). As a control, the nuclei were stained with DAPI for visualization under a fluorescence microscope (Nikon Eclipse C1). Afterward, the images were analyzed using the Image J software to calculate the MOD values for quantification. Meanwhile, histological examinations of these tumors were performed using hamatoxylin and eosin (H&E) staining method to detect the impact on the histology of tumor tissues. These measurements were repeated twice.

To evaluate the impact of D-bLP-mediated photothermia on cancer cells, the two-tumor model was developed using the 4T1-GFP cells, and then treated as described above. At certain intervals after laser irradiation, these tumors were cryosectioned and stained with DAPI. Thereafter, these sections were visualized under the fluorescence microscope (Nikon Eclipse C1), and the 4T1-GFP cells were denoted as clustered cells with green signals. The images were analyzed using the Image J software to calculate the MOD value for quantification. These measurements were repeated twice.

### Impact of TSM remodeling on tumor targeting of M-bLP

To clarify the possible impact of D-bLP-mediated TSM remodeling on tumor accumulation and penetration of second M-bLP, M-bLP was fluorescently labeled with DiI by physical entrapment to prepare the DiI/M-bLP, which would not interfere with the imaging signals of D-bLP. In the two-tumor model at 12 -h post injection of D-bLP, one tumor mass was irradiated at 808 -nm laser for 5 min, and the other one was not irradiated as a control. The second DiI/M-bLP was injected to the two-tumor model (3.0 mg·kg^−1^ of DiI) at 0, 2.0, and 4.0 h after laser irradiation (Supplementary Fig. 15) to optimize the injection time points. At 4 -h post injection, mice were autopsied and the major organs were collected for imaging. The measured results indicated that the second DiI/M-bLP had better to be injected immediately after laser irradiation.

In the following measurements, the two-tumor model was first treated with D-bLP or D-Lipo at 5.0 mg·kg^−1^ of DiR via tail vein. At 12 h after injection, one tumor mass was exposed to 808 -nm laser for 5 min, and the other one was not irradiated as a control. Immediately after the laser irradiation, DiI/M-bLP or the counterpart DiI/M-Lipo was injected to the two-tumor model at 3.0 mg·kg^−1^ of DiI. At 4.0 -h post injection, mice were anaesthetized, imaged under the in vivo imaging system, and then autopsied to collect the major organs including the heart, liver, spleen, lung, kidney, the laser-irradiated tumor, and control tumor for further imaging (*n* = 3). Then, the intensity of fluorescence signals in each tumor was analyzed by the accompanied software for quantification (*n* = 3).

Then, to determine the impact of D-bLP-mediated TSM remodeling on tumor penetration of DiI/M-bLP or DiI/M-Lipo, the laser-irradiated tumor mass and untreated control tumor were collected at 4.0 h of injection, then embedded in tissue optimal cutting temperature (OCT) compound for cryostat section (Leica 1950, Germany). The sectioned slices were stained with phalloidin-FITC (Beyotime, C1033, 1:100) and DAPI (Beyotime, C1002) for detection under LCSM. Afterward, the whole tumor mass was scanned under the LCSM (TCS-SP8 STED, Leica, Germany), and the fluorescence signals were recorded to assess the penetration of DiI/M-bLP and DiI/M-Lipo in tumor mass. Meanwhile, the presence of CAF and TAM in laser-irradiated tumor and untreated control tumor from each group was outlined by the aforementioned methods. By contrast, the sections were stained with DAPI for observations under LCSM.

### Impact on M-bLP extravasation from tumor vasculature

In the two-tumor model at 12 -h post injection of D-bLP, one tumor mass was irradiated at 808 -nm laser for 5 min, and the other one was not irradiated as a control. DiI/M-bLP was injected to the two-tumor model immediately after the laser irradiation at 3.0 mg·kg^−1^ of DiI. The extravasation of DiI/M-bLP from tumor vasculature was measured using a two-photon microscope (TCS SPS CFSMP, Leica, Germany). For the measurement, mice were intravenously injected with FITC-dextran (70000 Da, 46945, Sigma) to labeled the tumor vasculatures at 15 min prior to the removal of tumor mass. At 4.0 -h post injection of DiI/M-bLP, the laser-irradiated tumor and control tumor were collected, embedded in OCT compound, and sectioned at 100 µm. The sections were mounted onto slides and visualized under LCSM. The red signals of DiI/M-bLP and the green colors of tumor vessels were, respectively, recorded to evaluate the extravasation of M-bLP from tumor vasculature.

Then, the impact of D-bLP-mediated TSM remodeling on the diffusion of DiI/M-bLP from tumor vessel into the distal matrix was further evaluated. At 4.0 h after injection, the laser-irradiated tumor and control tumor were collected for cryostat section at 10 µm. Then, the sections were stained with anti-CD31 antibodies (Abcam, ab28364, 1:250) and secondary antibody of FITC-labeled IgG (H + L) (Beyotime, A0562, 1:400) for specific labeling of tumor vessels. The nuclei were stained with DAPI as a control. Thereafter, samples were monitored using LCSM (TCS-SP8 STED, Leica, Germany) to record the fluorescence signals of tumor vessels and DiI/M-bLP, respectively. The diffusion of DiI/M-bLP away from tumor vessels were analyzed by the Image J software. The intensity of fluorescence signals from DiI/M-bLP in three different directions away from the tumor vessels was calculated for the assessments (*n* = 3).

### TSM-remodeling promotes M-bLP accessibility to cancer cells

To elucidate the impact of D-bLP-mediated TSM remodeling on M-bLP accessibility to cancer cells, at 12 -h post injection of D-bLP in the two-tumor model, one tumor mass was irradiated and the other one was not irradiated as a control. DiI/M-bLP was injected to the two-tumor model immediately after the laser irradiation. By contrast, the counterpart liposomal formulations of D-Lipo and DiI/M-Lipo were performed in the same program. At 4.0 -h post injection of second DiI/M-bLP or DiI/M-Lipo, the laser-irradiated tumor mass and untreated control tumor from each group were collected, cryosectioned at 10 µm, and then, respectively, labeled to clarify the accessibility of second nanosystems to the cancer cells, CAF, and TAM in tumor using LCSM (TCS-SP8 STED, Leica, Germany).

In the 4T1-induced two-tumor model, the tumor sections were, respectively, labeled with specific antibodies against α-SMA (Abcam, ab5694, 1:500) and CD31 (Abcam, ab7388, 1:250), then followed with secondary antibodies of Alexa Fluor 647-labeled IgG (H + L) (Beyotime, A0468, 1:400) and Alexa Fluor 488-labeled IgG (H + L) (Yeasen, 33316ES60, 1:50) to characterize the CAF fractions in tumor (α-SMA^+^/CD31^−^ cells), which was depicted as cells with green signals, excluding white signals. Then, these sections were stained with specific antibodies of F4/80 (Abcam, ab6640, 1:250) and secondary antibodies of Alexa Fluor 488-labeled IgG (H + L) (Yeasen, 33316ES60, 1:50) to label the TAM fractions in tumor. The internalization of DiI/M-bLP or DiI/M-Lipo by CAF or TAM was denoted as the localization of red signals within these stromal cells. In addition, the CSC fractions in tumor were labeled with the typical antibody against ALDH1 (Abcam, ab52492, 1:250) and secondary antibody of FITC-labeled IgG (H + L) (Beyotime, A0562, 1:400) to detect the accessing of second M-bLP to the ALDH^high^ CSCs in tumor. By contrast, the nuclei were stained with DAPI for the visualization. These measurements were repeated twice.

Importantly, to determine the impact of TSM remodeling on nanoparticles accessibility to cancer cells, the group of D-bLP following with DiI/M-bLP as well as the counterpart liposomal group of D-Lipo following with DiI/M-Lipo were successively injected to the 4T1-GFP-induced two-tumor model as described above. At 4.0 h after injection of DiI/M-bLP or DiI/M-Lipo, the laser-irradiated tumor and untreated control tumor were collected and cryosectioned at 10 µm. By contrast, the nuclei were stained DAPI for visualization under LCSM. The accessibility of DiI/M-bLP or DiI/M-Lipo to cancer cells was denoted as the co-localization of red signals with 4T1-GFP cancer cells. Moreover, the localization of DiI/M-bLP in the regions of 4T1-GFP cell clusters was analyzed using the Image J software. The gray values between two white lines were calculated to evaluate the accessibility of M-bLP to cancer cells in tumor mass. These measurements were repeated twice.

### In vitro therapeutic efficacy in metastatic 4T1 cells

To evaluate the in vitro therapeutic efficacy, cellular uptake of D-bLP by 4T1 cells, CAF, and TAM were, respectively, visualized under LCSM (TCS-SP8 STED, Leica, Germany). The primary CAF were isolated from 4T1-induced tumor mass by a repeated adherence method^61,62^. In brief, the isolated tumor tissues were cut into small pieces, and followed by digestion with collagenase (type I, 1.0 mg·mL^−1^, Yeasen) and hyaluronidase (1.0 mg·mL^−1^, Yeasen) in sterile serum-free DMEM media (penicillin, 100 U·mL^−1^, streptomycin 100 µg·mL^−1^) at 37 °C and 5% CO_2_ in a humidified incubator for 8 h. Then, the suspension was filtered through a 70-μm stainless mesh to prepare single-cell suspension. The filtrate was centrifuged, washed twice with DMEM media, re-suspended in DMEM media with 10% FBS, and incubated in culture dishes for 12 h. The unadherent cells were removed, and the adherent cells were cultured in fresh DMEM media for further 48 h. Then, these cells were digested, suspended in serum-free DMEM media, and plated onto culture dishes for 5 min to obtain the adherent CAF. These adherent CAF were purified by this repeated adherence method, and characterized by typical markers of α-SMA and CD31 (α-SMA^+^/CD31^−^) for further measurements. RAW 264.7 cells were used as a typical cell line of TAM. The cellular uptake of D-bLP was measured in single-cell phenotype model and mixed-cell phenotype model. For flow-cytometer analysis, the gating strategies of these cell models were documented (Supplementary Figs. 39, 40) for further measurements.

To evaluate the uptake of D-bLP by each cell phenotype, these cells were, respectively, seeded on the round glass coverslips (Ø 10 mm) in 24-well plate at 60,000 cells/well and cultured overnight. Then, D-bLP was added to each well at 10 µg mL^−1^ of DiR and incubated for 4 h. Afterward, cells were counterstained with phalloidin-FITC (Beyotime, C1033, 1:100) and Hoechst 33342 (Beyotime, C1027, 1:100) for visualization under LCSM. Moreover, the cellular uptake of D-bLP was determined by flow cytometer for quantification.

To detect the preferential cellular internalization of D-bLP in the mixed-cell phenotype model, the 4T1 cancer cells (or 4T1-GFP cell), CAF, and TAM were mixed at 1:1:1 and incubated overnight. The mixed cells were incubated with D-bLP at 10 µg mL^−1^ of DiR for 4 h for further measurements. To investigate the uptake by 4T1 cancer cells, 4T1-GFP cells were mixed with isolated CAF and RAW 264.7. After 4 h of incubation, the internalization of D-bLP by 4T1-GFP cells were visualized under LCSM and quantified by flow-cytometer analysis. The ratio of 4T1-GFP cancer cells, TAM (RAW 264.7), and CAF (α-SMA^+^) in the mixed-cell model were analyzed by flow cytometer to monitor the measurements (Supplementary Fig. 23). By contrast, the nuclei were stained with Hoechst 33342 (Beyotime, C1027, 1:100) for LCSM measurements.

To clarify the uptake of D-bLP by CAF or TAM, CAF, TAM, and 4T1 cancer cells were mixed at 1:1:1 and cultured overnight. After 4 h of incubation with D-bLP at 10 µg mL^−1^ of DiR, cells were stained with the primary antibody against α-SMA (Abcam, ab5694, 1:10) and FITC-labeled secondary antibody (Beyotime, A0562, 1:100) to outline CAF. By contrast, the nuclei were stained with Hoechst 33342 (Beyotime, C1027, 1:100) for imaging under LCSM. Meanwhile, these cells were stained with Alexa Fluor 488-labeled antibody of α-SMA (Novus Biologicals, NBP2–34522AF488, 1:20) for flow-cytometer analysis. To elucidate the uptake of D-bLP by TAM, cells were stained with the primary antibody against F4/80 (Abcam, ab6640, 1:10) and Alexa Fluor 488-labeled secondary antibody (Yeasen, 33316ES60, 1:10) for LCSM visualization. By contrast, the nuclei were stained with Hoechst 33342 (Beyotime, C1027, 1:100) for the measurements. Meanwhile, these cells were stained with PE anti-mouse F4/80 (Biolegend, 123110, 1:20) for flow-cytometer analysis. The measurements were performed in triplicate.

To elucidate the possible mechanism of the preferential uptake of D-bLP by CAF and TAM over 4T1 cancer cells, the expression of SR-BI and CD36 in these cells was determined by flow-cytometer analysis. To detect the SR-BI expression, CAF, TAM, and 4T1 cancer cells were, respectively, stained with the primary anti-SR-BI antibody (Abcam, ab217318, 1:10), and incubated with secondary antibody of Alexa Fluor 647-labeled IgG (H + L) (Beyotime, A0468, 1:100) for the analysis. To detect the expression of CD36, these cells were, respectively, incubated with primary antibody of anti-CD36 (Abcam, ab80080, 1:10) and followed with the secondary antibody of Alexa Fluor 488-labeled IgG (H + L) (Yeasen, 33316ES60, 1:10) for the measurements. To quantify the impact of these receptors on the cellular uptake of D-bLP, cells were, respectively, pretreated with specific antibodies of CD36 (Abcam, ab80080, 1:20), SR-BI (Abcam, ab217318, 1:20), their combinations or absence (control group), and then incubated with D-bLP for 4 h for analysis by flow cytometer. By contrast, the untreated cells without D-bLP incubation were used as negative control. The measurements were performed in triplicate.

Then, the therapeutic effects of D-bLP + laser, M-bLP, and their synergistic combination on cell viability, migration, and stemness were measured in metastatic 4T1 cells. In brief, cells were seeded in 96-well plate at 4000 cells/well and cultured overnight. For D-bLP + laser, D-bLP was added to cells at 10 µg·mL^−1^ or 0.1 µg·mL^−1^ of DiR, and further incubated for 4 h. Then, the media was replaced with fresh culture media, and cells were exposed to 808 -nm laser at 2.0 W·cm^–2^ for 3 min. The laser-irradiated cells were further cultured for 48 h for the assays. For M-bLP, cells were incubated with M-bLP at equivalent 1.0 µg·mL^−1^ or 0.01 µg·mL^−1^ of mertansine, and then cultured for 48 h for the assays. For their synergistic combination of D-bLP + laser/M-bLP, cells were first incubated with D-bLP at 10 µg·mL^−1^ or 0.1 µg·mL^−1^ of DiR for 4 h, replaced with fresh culture media, irradiated at 808 -nm laser at 2.0 W·cm^–2^ for 3 min, and then incubated with M-bLP at equivalent 1.0 µg·mL^−1^ or 0.01 µg·mL^−1^ of mertansine for further 48 h. Meanwhile, cells without any treatment or with laser irradiation alone were performed as negative control. Afterward, cell viability was measured by MTT assay (Enspire, PerkinElmer, Singapore). The measurements were performed in triplicate.

Next, the anti-migration activities of these groups were measured using transwell assays. The pretreated cells from each group were added to the upper chambers of inserts (24-well, pore size, 8 µm, Costar) at 2 × 10^5^ cells per well in 200 µL of serum-free culture media. Then, 500 µL of 10% FBS containing culture media were added to each well of 24-well plate. After 24 h, the migrated cells across the transwell membrane were stained with crystal violet and imaged under a microscope (IX81, Olympus, Japan). The migration rate was defined as the percentage of migrated cells in each group compared with that in negative control. The measurements were performed in triplicate.

Moreover, to assess impact of these treatment on the stemness of residual cancer cells, these pretreated cancer cells in control, laser alone, D-bLP + laser (10 µg·mL^−1^ of DiR), M-bLP (1.0 µg·mL^−1^ of mertansine), and D-bLP + laser/M-bLP (10 µg·mL^−1^ of DiR and 1.0 µg·mL^−1^ of mertansine) were stained with the Aldefluor^TM^ Kit (STEMCELL Technologies, Canada), and then analyzed by a flow cytometer to characterize the ratio of ALDH^high^ cells. Meanwhile, the un-stained cells from each group were performed as the gate of ALDH^high^ cells. These measurements were repeated twice.

Thereafter, the tumorsphere-forming assays were used to characterize the self-renewal ability of these residual cancer cells. The pretreated cancer cells in each group were added to a six-well plate at 4 × 10^4^ cells per well and cultured with serum-free DMEM/F12 culture media supplemented with 5 µg·mL^−1^ of insulin, 20 ng·mL^−1^ of epidermal growth factor (EGF), 20 ng·mL^−1^ of basic fibroblast growth factor (bFGF), 1 × B27 (Invitrogen, USA), 0.4% (w/v) bovine serum albumin, 100 U·mL^−1^ of penicillin, and 100 µg·mL^−1^ of streptomycin. After 4 days, the formation of tumorsphere in each group was visualized under the inverted microscope (IX81, Olympus, Japan).

### In vivo therapeutic effects on tumor relapse and metastasis

The therapeutic effects of bLP-mediated TECA strategy on tumor relapse and metastasis were evaluated in two breast cancer tumor models. The murine 4T1 metastatic tumor model was induced by injecting 4T1 cells to the fourth mammary pad of nude mice at 1 × 10^6^ cells per mouse. When the tumor volume reached 100 mm^3^, mice were randomly divided into 12 groups (*n* = 6), and, respectively, treated with saline control, laser alone, D-bLP, D-bLP + laser, M-bLP, M-bLP + laser, DM-bLP + laser, D-bLP + laser/M-bLP and the liposome counterparts of D-Lipo + laser, M-Lipo, DM-Lipo + laser, and D-Lipo + laser/M-Lipo. For laser-irradiated groups, mice were anaesthetized at 12 -h post injection of D-bLP or D-Lipo, and tumors were performed single treatment of irradiation upon an 808 -nm laser at 2.5 W·cm^–2^ for 5 min. The thermal profiles and surface temperature of tumor mass were recorded using the infrared thermal camera (A150–15-M, Irtech Ltd). In laser alone, the D-bLP + laser and D-Lipo + laser group, no additional treatments were performed after the first laser irradiation treatment. In M-bLP + laser, DM-bLP + laser, D-bLP + laser/M-bLP, liposomal DM-Lipo + laser, and D-Lipo + laser/M-Lipo-treated groups, these mertansine-loaded nanosystems were injected to the tumor model at 0.5 mg·kg^−1^ of mertansine twice a week after the laser irradiation treatment. In D-bLP, M-bLP and M-Lipo groups, mice were treated at 0.5 mg·kg^−1^ of mertansine or 5.0 mg·kg^−1^ of DiR twice a week. During these treatment, the body weights in each group were supervised, and the tumor size was monitored using a digital caliper to calculate the tumor volumes. At day 20 after the first treatment, mice were autopsied to collect the tumor and lung tissues in each group. The tumor mass from each group was photographed and weighed to calculate the inhibitory effects on tumor growth and relapse. Meanwhile, the visualized metastatic nodules in lungs from each group were recorded to calculate the therapeutic effects on lung metastasis. Thereafter, the lungs from each group were embedded in the paraffin, sectioned at 5 μm, and stained with the H&E staining kit for histopathological analysis.

Likewise, the therapeutic effects of D-bLP + laser/M-bLP were also evaluated in a MCF-7-induced human breast cancer tumor xenografts. The tumor models were induced by injecting MCF-7 cells to the fourth mammary pad of nude mice at 5 × 10^6^ cells per mouse. When the tumor volumes reached about 150 mm^3^, mice were, respectively, treated with saline control, laser alone, D-bLP + laser, M-bLP, D-bLP + laser/M-bLP, and the liposome counterpart of D-Lipo + laser/M-Lipo as aforementioned programs (*n* = 5). The body weight, tumor relapse after therapy, and the tumor volumes in each group were monitored during these treatments. At day 25 after the treatments, the tumor tissues from each group were collected and weighed to calculate the tumor inhibitory rate.

### Statistical analysis

The data were presented as mean ± standard deviation (SD). For a two-group comparison, a Student's *t* test was performed for the statistical analysis. For multiple comparisons, the data were analyzed using analysis of variance (ANOVA). Significant difference was considered when the *p*-value was less than 0.05.

### Reporting summary

Further information on research design is available in the Nature Research Reporting Summary linked to this article.

## Supplementary information

Supplementary information

Reporting Summary

Source Data

## Data Availability

The data supporting the findings of this study are available within this paper and Supplementary information. Source data of Figs. 2c-e, 3c, 4e-f, 5d, 6c, 7e, 8c-e, 9b-f, and Supplementary Figs. 2, 3, 14, 20, 24–27, 29, 30b, 32 a-d, 33, 35, 36 are provided as a Source data file. Additional data can also be available from the corresponding author on reasonable request.
