# Supplementary material for: Bioinspired lipoproteins-mediated photothermia remodels tumor stroma to improve cancer cell accessibility of second nanoparticles
Source: Nat Commun. 2019 Jul 25;10:3322. doi: 10.1038/s41467-019-11235-4 (PMC6658501; doi:10.1038/s41467-019-11235-4)
Supplement: Supplementary file 1 — Supplementary information [file 41467_2019_11235_MOESM1_ESM.pdf]

Supplementary Information

Bioinspired lipoproteins-mediated photothermia remodels tumor stroma to improve cancer cell accessibility of second nanoparticles

Tan et al

Supplementary Figures

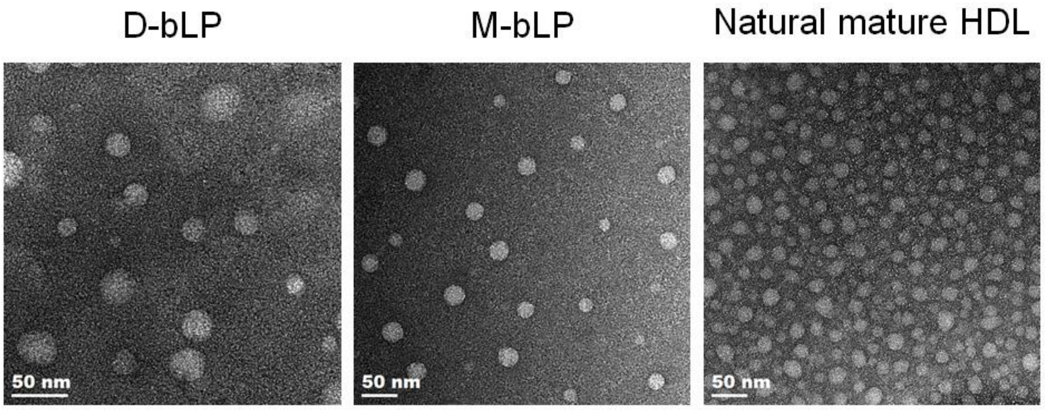

Supplementary Figure 1 Typical TEM images of D-bLP, M-bLP and natural mature HDL, scale bar, 50 nm. The statistical analysis showed the mean diameters of D-bLP, M-bLP and natural mature HDL from human serum (Sigma, L1567) were 23.6±4.8 nm, 26.4±5.2 nm and 13.2±2.8 nm, respectively.

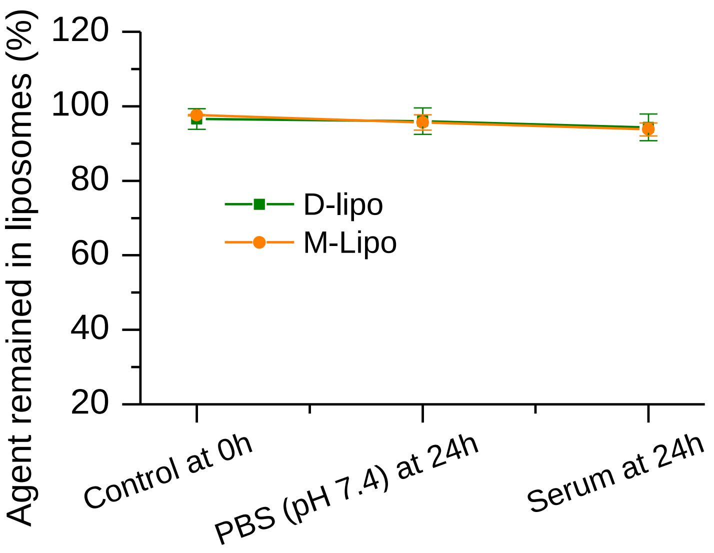

Supplementary Figure 2 The percentage of DiR or mertansine remained in the liposomal formulations of D-Lipo or M-Lipo after 24 h of incubation in PBS (pH 7.4) or fetal bovine serum. Data are means ± SD, n=3.

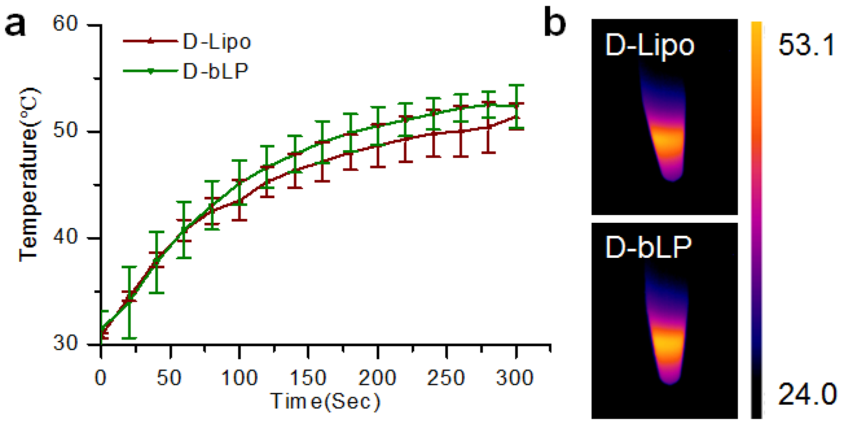

Supplementary Figure 3 The temperature changes (**a**) and typical thermal images (**b**) of D-Lipo and D-bLP upon their exposure to 808 nm laser for 5 min. Data are means ± SD, n=3.

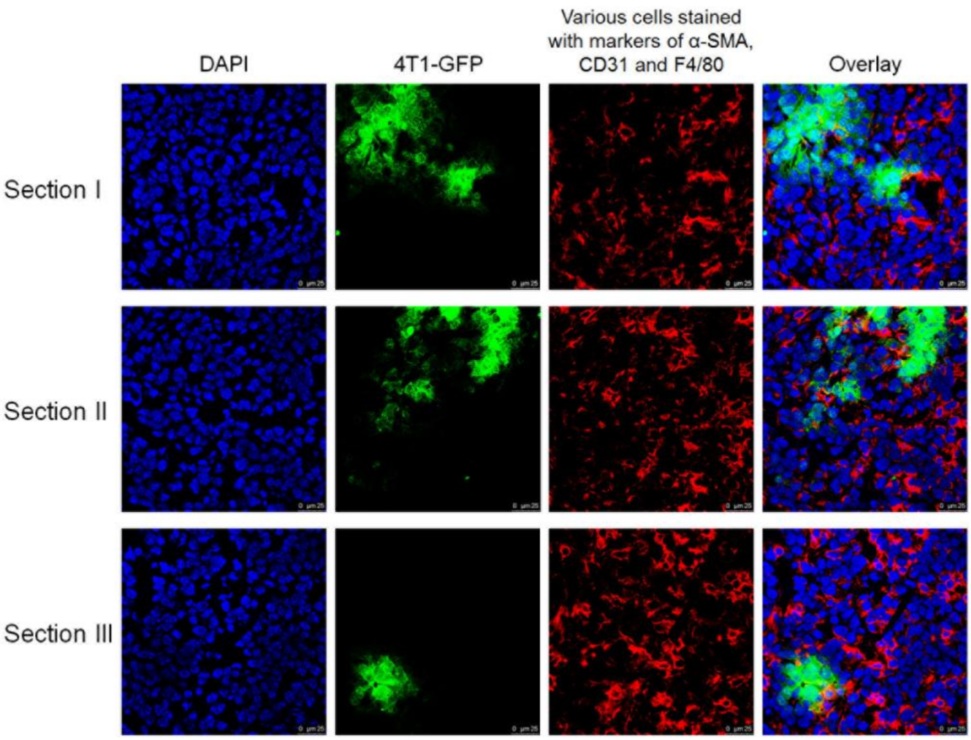

Supplementary Figure 4 The distribution of 4T1-GFP cancer cells (green signals) and different stromal cells (red signals of TAM, CAF and EC) in different tumor sections, scale bar, 25 μm. The tumor model was induced with 4T1-GFP cells for the detection. The tumor sections were respectively stained with specific antibodies against α-SMA, CD31 and F4/80, and then followed with Cy3-labeled secondary antibodies to outline these cells in the same tumor section. The tumor microenvironments were mainly composed of cancer cells, CAF, TAM, EC and various immune cells, etc. In the captured images from different tumor sections, 4T1-GFP cells, TAM, CAF and EC account for the vast majority of the cells in tumor. The small amount of unlabeled cells would be some immune cells that have infiltrated in the tumor mass.

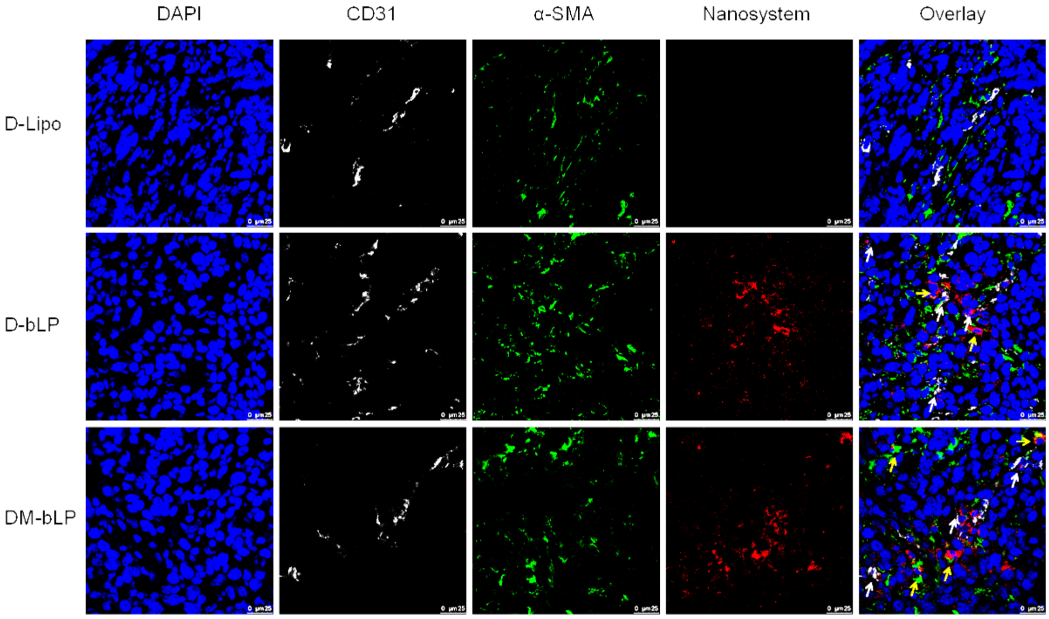

Supplementary Figure 5 The in vivo internalization of D-Lipo, D-bLP and DM-bLP by CAF in tumor regions. CAF were characterized by α-SMA positive and CD31 negative cells (α-SMA^+/$CD31^-$), which was denoted as cells with green signals excluding white signals. By contrast, cells with white signals were presented as EC of tumor vessels, scale bar, 25 μm. The yellow arrows referred to the internalization of various nanosystems by CAF, while the white arrows meant the uptake by EC cells.

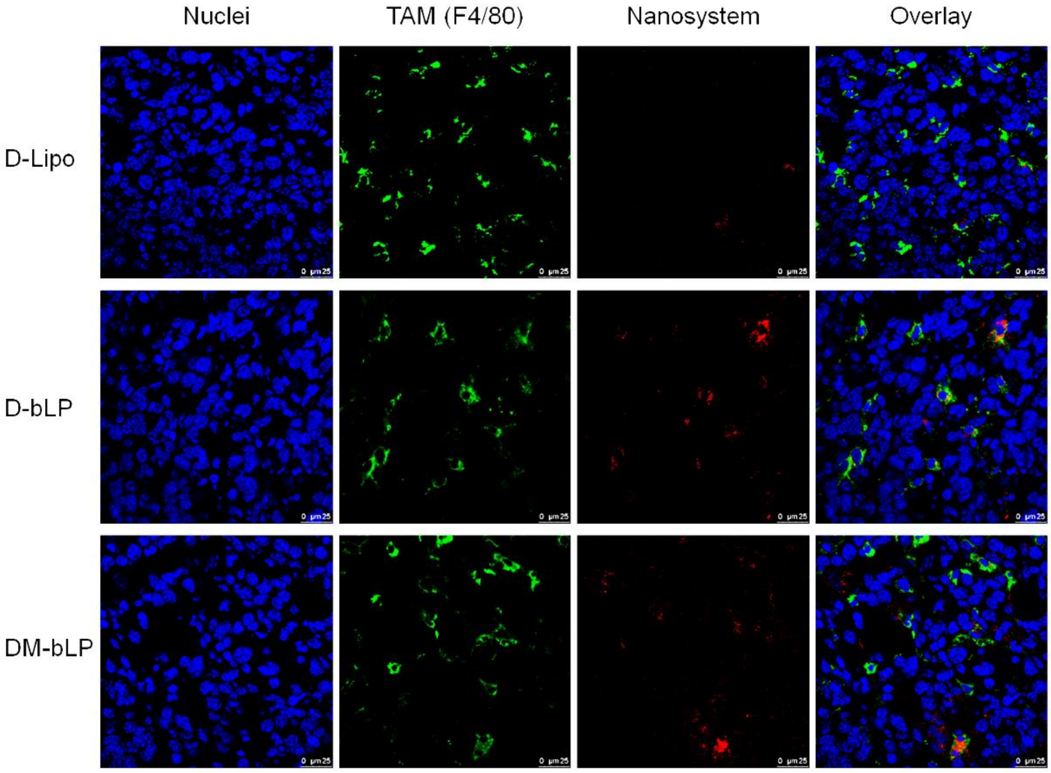

Supplementary Figure 6 The in vivo internalization of D-Lipo, D-bLP and DM-bLP by TAM in tumor. TAM were characterized by F4/80 positive cells in the captured images (green signals), scale bar, 25 μm.

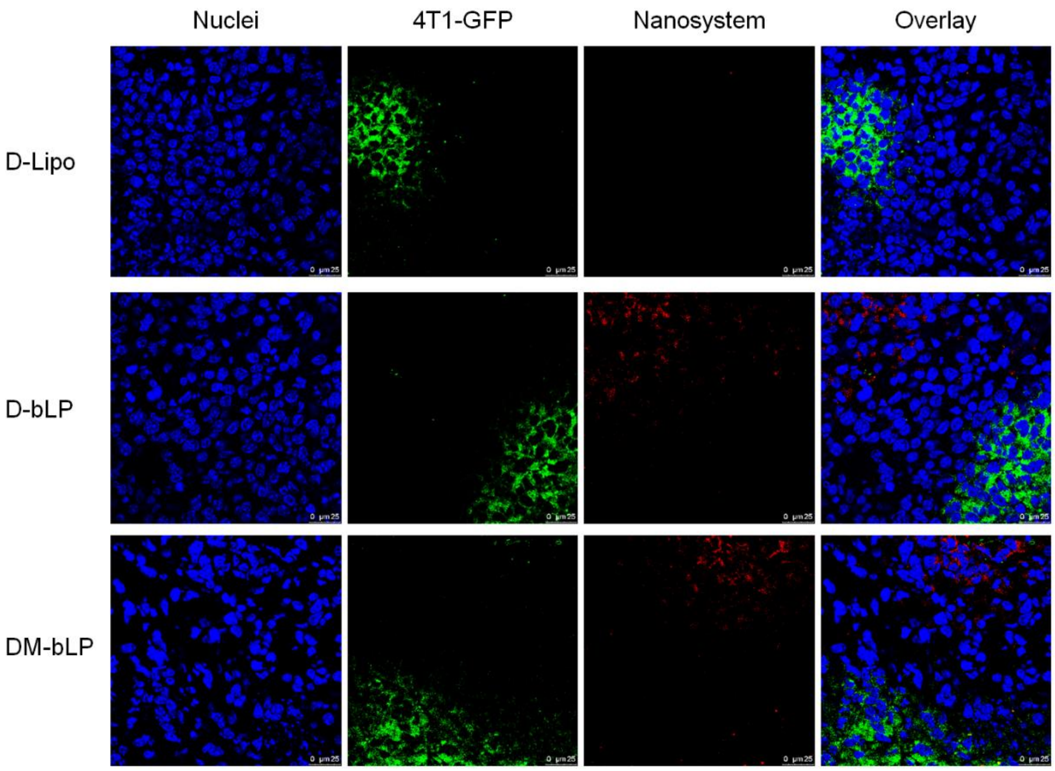

Supplementary Figure 7 The accessibility of D-Lipo, D-bLP and DM-bLP to 4T1-GFP cells regions in tumor. The 4T1-GFP cells were denoted as green signals in the captured images, scale bar, 25 μm.

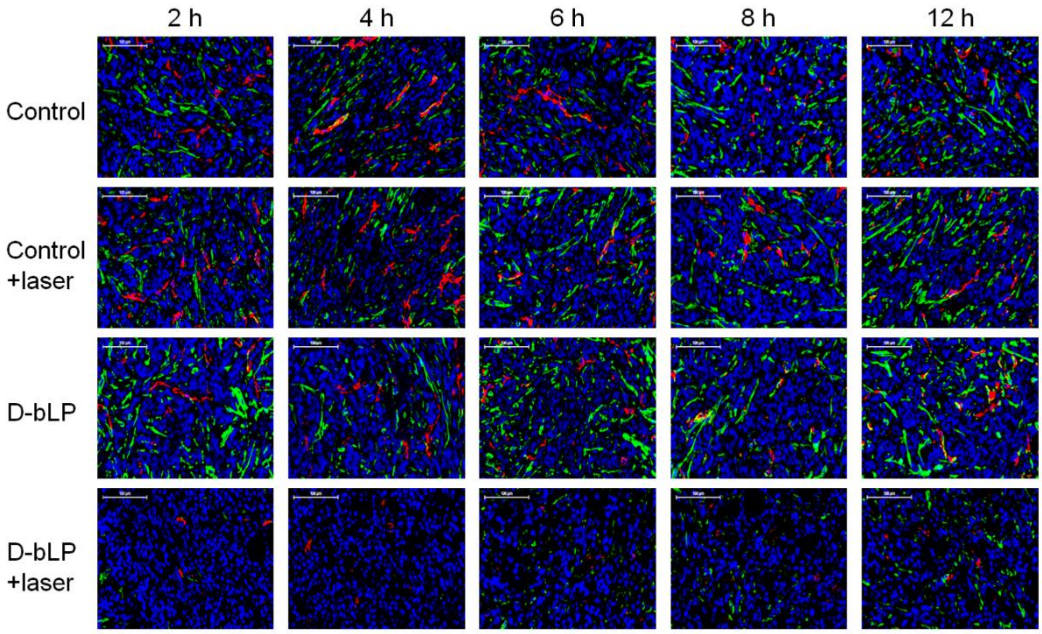

Supplementary Figure 8 The proportion of CAF and EC in tumor at different time points after laser irradiation in control mice or D-bLP treated group. In 4T1-induced two-tumor model, one tumor mass was irradiated with an 808 nm laser at 2.5 W cm^{-2} for 5 min, and the other one was not irradiated as control. The laser irradiation was performed at 12 h after D-bLP injection. By contrast, the tumor model without D-bLP injection were also performed in the same program. CAF were denoted as the green signals of α-SMA excluding the red signals of CD31 (α-SMA^{+}/$CD31^{-}$), while EC of tumor vessels were referred as cells with red signals, scale bar, 100 μm.

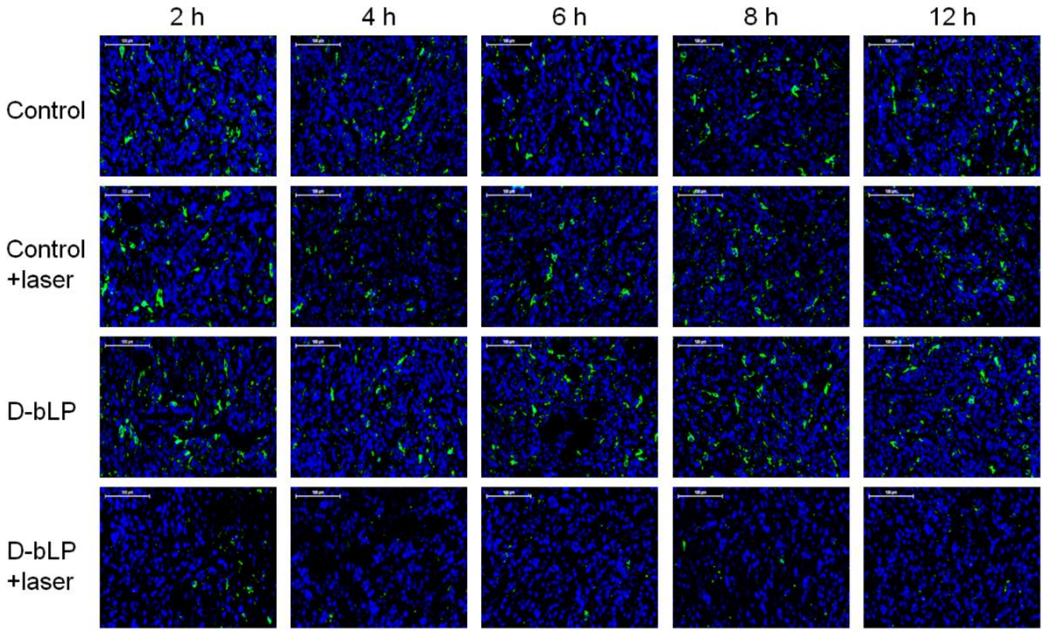

Supplementary Figure 9 The proportion of TAM in tumor regions at different time points after laser irradiation in control mice or D-bLP treated group. In 4T1-induced two-tumor model, one tumor mass was irradiated with an 808 nm laser at 2.5 W cm^{-2} for 5 min, and the other one were not irradiated. The laser irradiation was performed at 12 h after D-bLP injection. By contrast, the tumor model without D-bLP injection were also performed in the same program. TAM were denoted as F4/80 positive cells with green signals, scale bar, 100 µm.

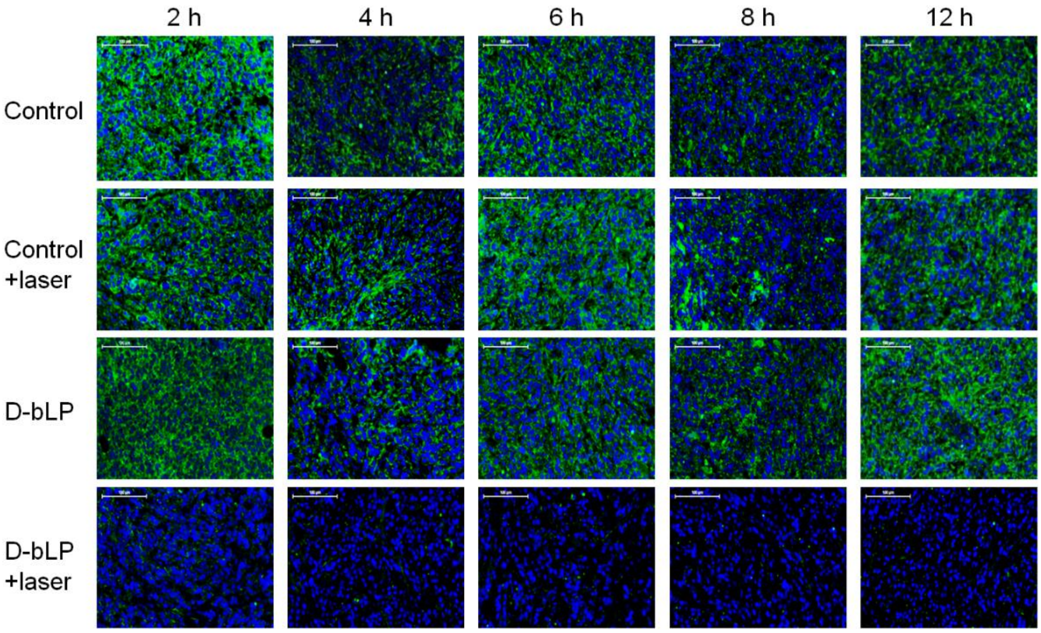

Supplementary Figure 10 The expression of collagen I in tumor regions at different time points after laser irradiation in control mice or D-bLP treated group. In 4T1-induced two-tumor model, one tumor mass was irradiated with an 808 nm laser at 2.5 W cm^{-2} for 5 min, and the other one were not irradiated as control. The laser irradiation was performed at 12 h after D-bLP injection. By contrast, the tumor model without D-bLP injection were also performed in the same program. Collagen I was expressed as green signals in the captured images, scale bar, 100 μm.

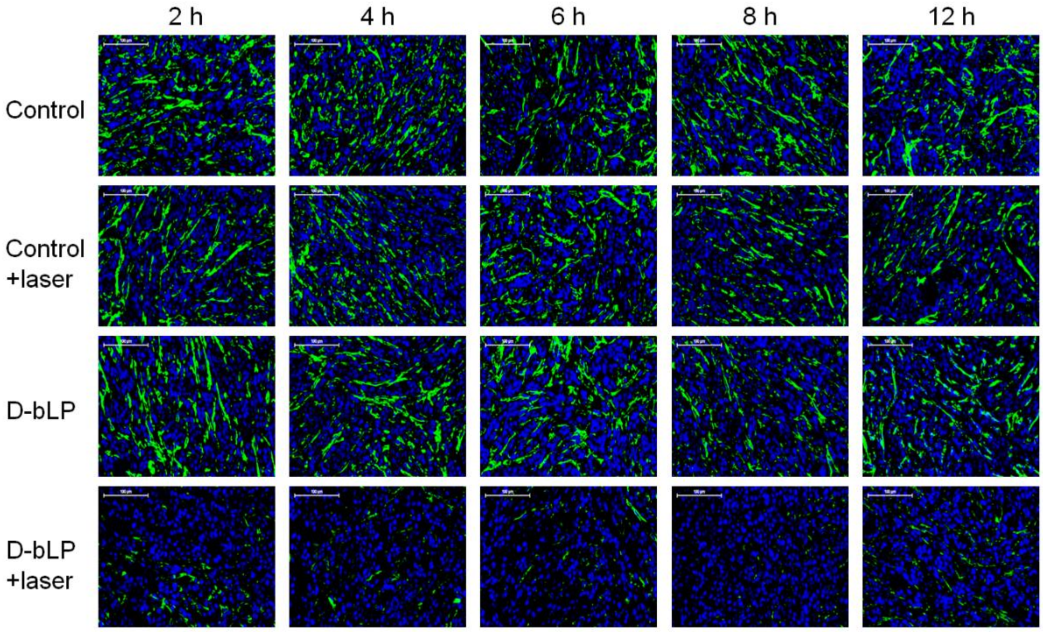

Supplementary Figure 11 The expression of fibronectin in tumor regions at different time points after laser irradiation in control mice or D-bLP treated group. In 4T1-induced two-tumor model, one tumor mass was irradiated with an 808 nm laser at 2.5 W cm^{-2} for 5 min, and the other one were not irradiated as control. The laser irradiation was performed at 12 h after D-bLP injection. By contrast, the tumor model without D-bLP injection were also performed in the same program. Fibronectin was presented as green signals in the captured images, scale bar, 100 μm.

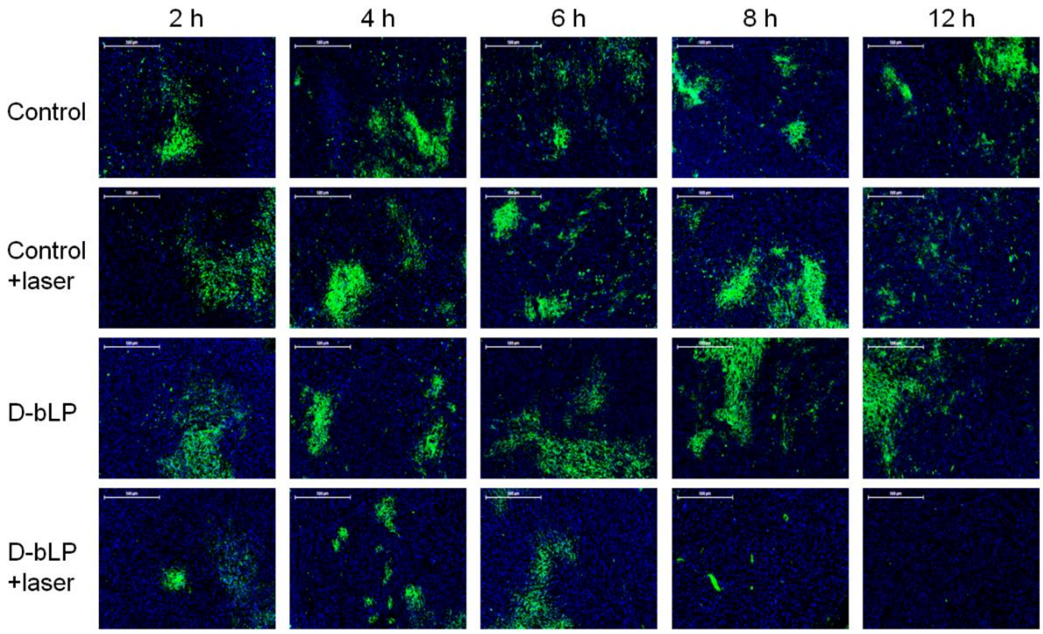

Supplementary Figure 12 The proportion of 4T1-GFP cancer cells in tumor regions at different time points after laser irradiation in control mice or D-bLP treated group. In the 4T1-GFP induced two-tumor model, one tumor mass was irradiated with an 808 nm laser at 2.5 W cm^{-2} for 5 min, and the other one were not irradiated as control. The laser irradiation was performed at 12 h after D-bLP injection. By contrast, the tumor model without D-bLP injection were also performed in the same program. In the captured images, 4T1-GFP cells were denoted as green signals, scale bar, 500 μm.

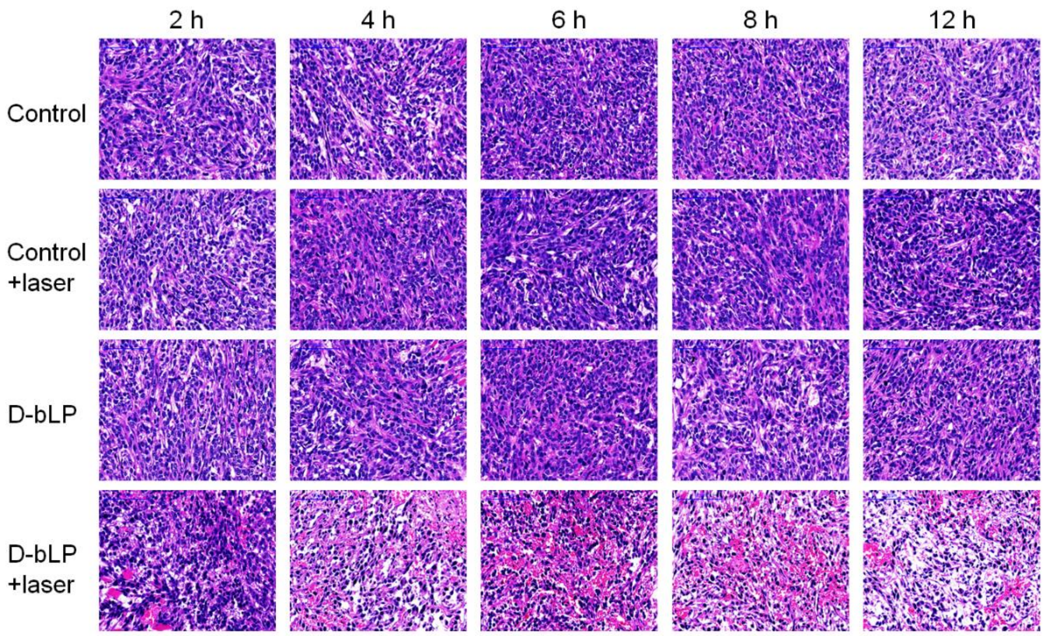

Supplementary Figure 13 Histological examinations of tumors at different time points after laser irradiation in control mice or D-bLP treated group. In the 4T1 induced two-tumor model, one tumor mass was irradiated with an 808 nm laser at 2.5 W cm^{-2} for 5 min, and the other one were not irradiated as control. The laser irradiation was performed at 12 h after D-bLP injection. By contrast, the tumor model without D-bLP injection was performed in the same program. At different time points after laser irradiation, tumor sections were measured by HE staining techniques. Cells in D-bLP+laser tumor became loosely packed with extensive karyopyknosis, hemorrhage and necrosis when comparing to other control tumors, scale bar, 100 μm.

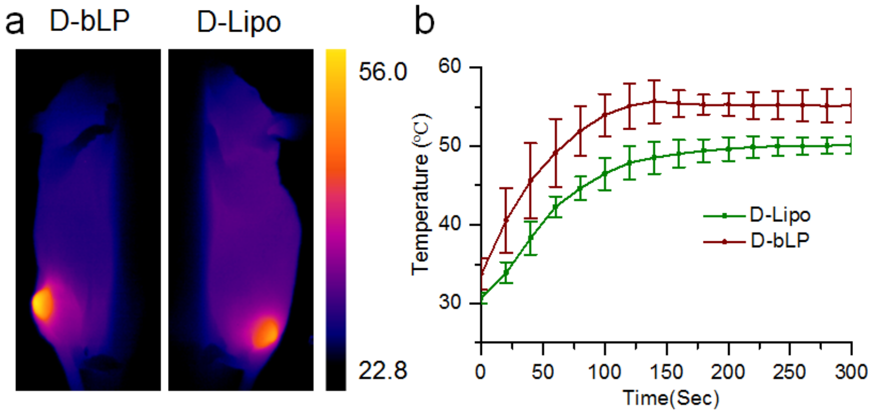

Supplementary Figure 14 The typical thermal images (**a**) and temperature changes (**b**) in tumor from 4T1-induced two tumor model treated with D-Lipo and D-bLP. At 12 h postinjection, one tumor mass was exposed to 808 nm laser at 2.5 W cm^{-2} for 5 min and the other one was not irradiated as control. The surface temperature of tumor with laser irradiation was recorded. Data are means ± SD (n=3).

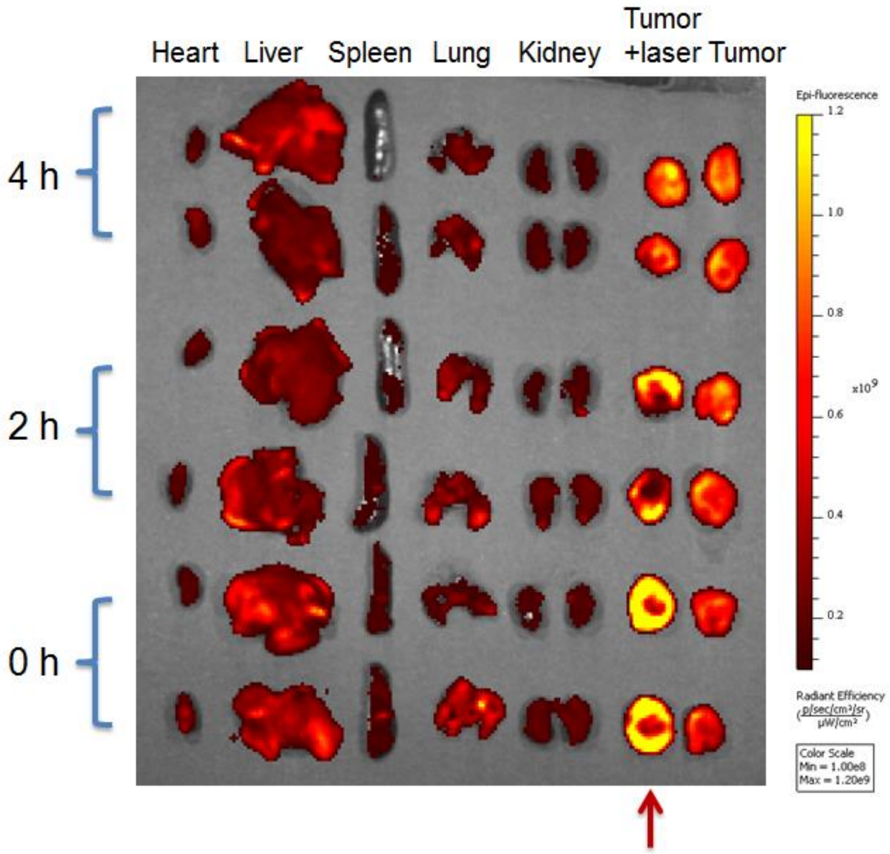

Supplementary Figure 15 The ex vivo distribution of second-delivered DiI/M-bLP in major organs. DiI/M-bLP was intravenously injected to the two-tumor model at 0 h, 2.0 h and 4.0 h after laser irradiation (n=2) (3.0 mg·kg^{-1} of DiI). By comparing the fluorescence signals in laser-irradiated tumor and untreated control tumors, DiI/M-bLP had better to be injected immediately after the laser irradiation. Red arrow refers to the tumor tissues with laser irradiation.

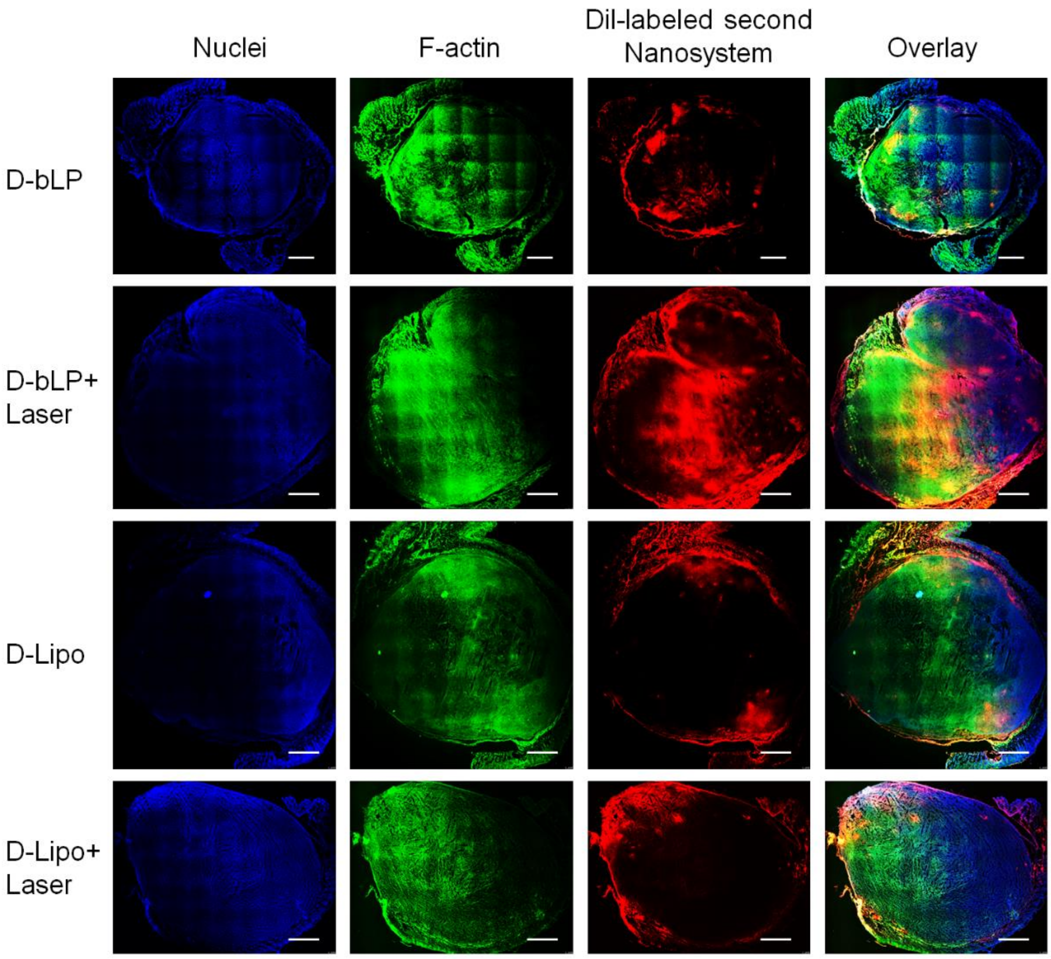

Supplementary Figure 16 Impact of D-bLP or D-Lipo mediated TSM-remodeling on the tumor penetration of second DiI/M-bLP or DiI/M-Lipo. The 4T1-induced two-tumor models were respectively injected with D-bLP and D-Lipo. At 12 postinjection, one tumor was exposed to 808 nm laser at 2.5 W cm^{-2} for 5 min and the other one was not irradiated as control. Immediately after the laser irradiation, DiI/M-bLP or DiI/M-Lipo was injected to the tumor model at 3.0 mg·kg^{-1} of DiI vail tail vein. After 4 h, the tumor tissues from each group were collected for visualization under LCSM. By contrast, sections were stained with phalloidin-FITC (Beyotime, C1033, 1:100) and DAPI (Bytotime, C1002) for the measurements. scale bar, 1.0 mm.

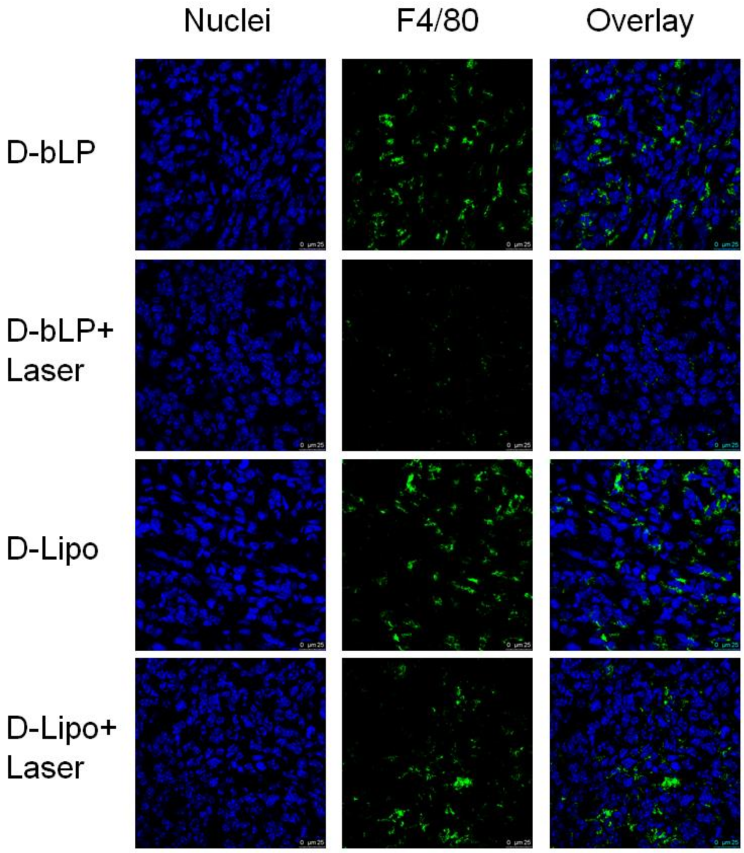

Supplementary Figure 17 Impact of D-bLP and counterpart D-Lipo mediated photothermal effects on TAM in tumor, scale bar, 25 μm. The 4T1-induced two-tumor models were respectively injected with D-bLP and D-Lipo. At 12 postinjection, one tumor was exposed to 808 nm laser at 2.5 W cm^{-2} for 5 min and the other one was not irradiated as control. Immediately after the laser irradiation, DiI/M-bLP or DiI/M-Lipo was injected to the tumor model at 3.0 mg·kg^{-1} of DiI vail tail vein. After 4 h, the tumor tissues from each group were collected and stained with specific antibodies for visualization under LCSM. TAM were denoted as green signals in the captured images. The expression of TAM was remarkably reduced by D-bLP mediated photothermal effects but mildly impacted by D-Lipo mediated photothermia.

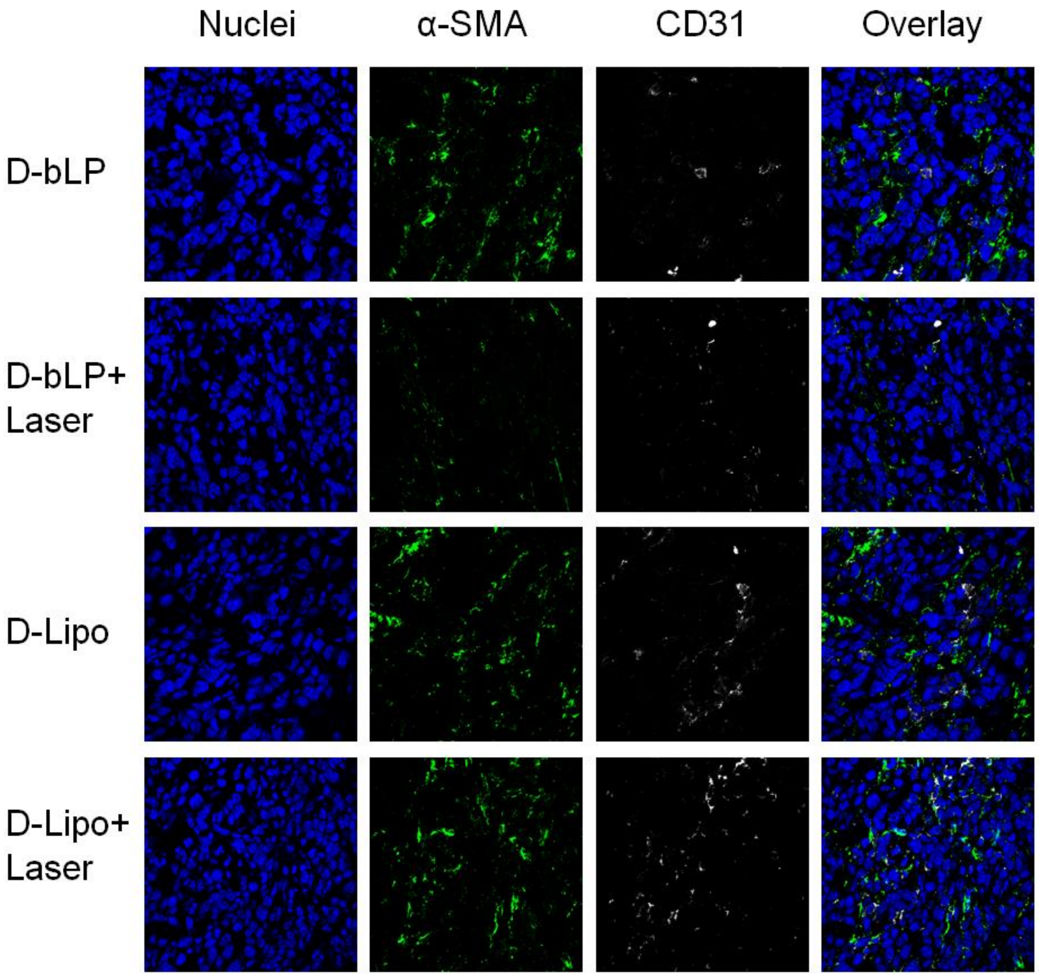

Supplementary Figure 18 Impact of D-bLP and counterpart D-Lipo mediated photothermal effects on CAF in tumor, scale bar, 25 μm. The 4T1-induced two-tumor models were respectively injected with D-bLP and D-Lipo. At 12 postinjection, one tumor was exposed to 808 nm laser at 2.5 W cm^{-2} for 5 min and the other one was not irradiated as control. Immediately after the laser irradiation, DiI/M-bLP or DiI/M-Lipo was injected to the tumor model at 3.0 mg·kg^{-1} of DiI vail tail vein. After 4 h, the tumor tissues from each group were collected and stained with specific antibodies for visualization under LCSM. CAF were characterized by α-SMA^{+}/$CD31^{-}$ cells, which were denoted as cells with green signals excluding white signals. By contrast, EC was presented as cells with white signals.

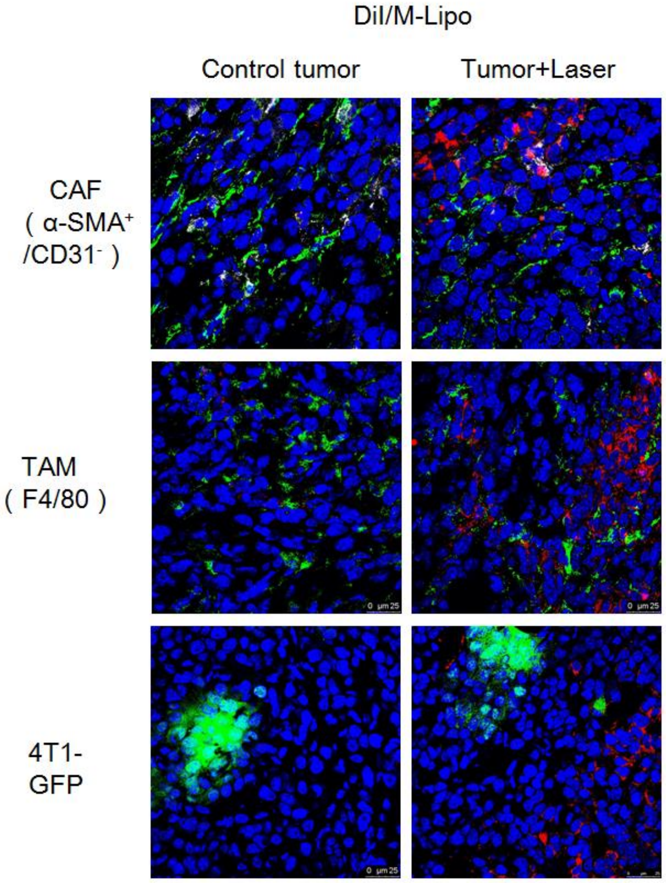

Supplementary Figure 19 Impact of counterpart D-Lipo mediated TSM-remodeling on the cellular uptake of second DiI/M-Lipo by CAF, TAM and 4T1-GFP cancer cells in tumor. CAF were characterized by α-SMA positive but CD31 negative cells (α-$SMA^{+}/CD31^{-}$). TAM and 4T1-GFP cancer cells were denoted as cells with green signals, scale bar, 25μm.

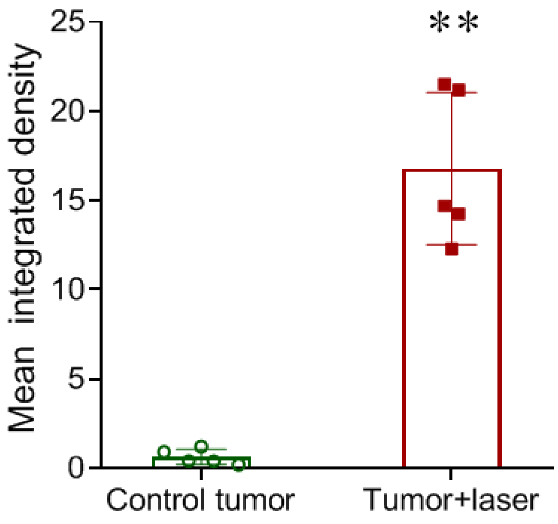

Supplementary Figure 20 The mean integrated density of DiI/M-bLP in 4T1-GFP cancer cells regions of tumor with and without laser irradiation (n=5). Data are means ± SD, **p<0.01 (two-tailed Student's t-test).

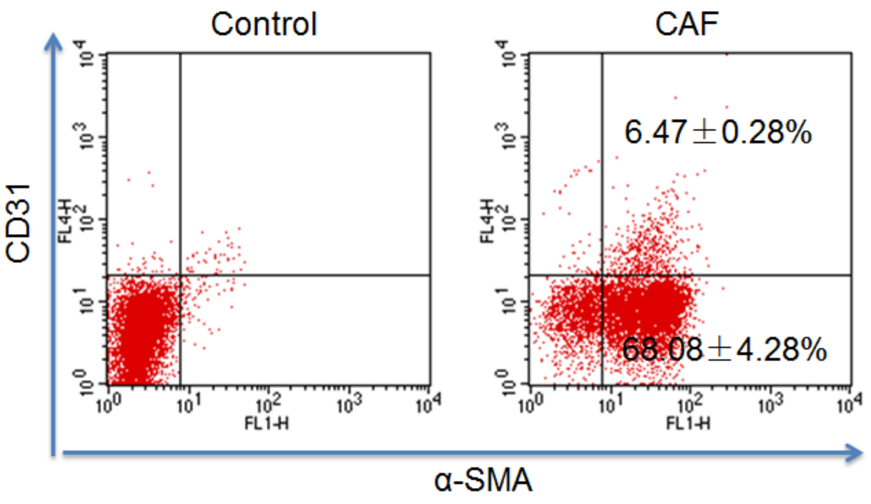

Supplementary Figure 21 Characterization of isolated CAF from 4T1-induced tumor mass by flow cytometer analysis. CAF was characterized by α-SMA positive and CD31 negative cells (α-SMA^+/$CD31^-$). The proportion of CAF was 68.08±4.28%.

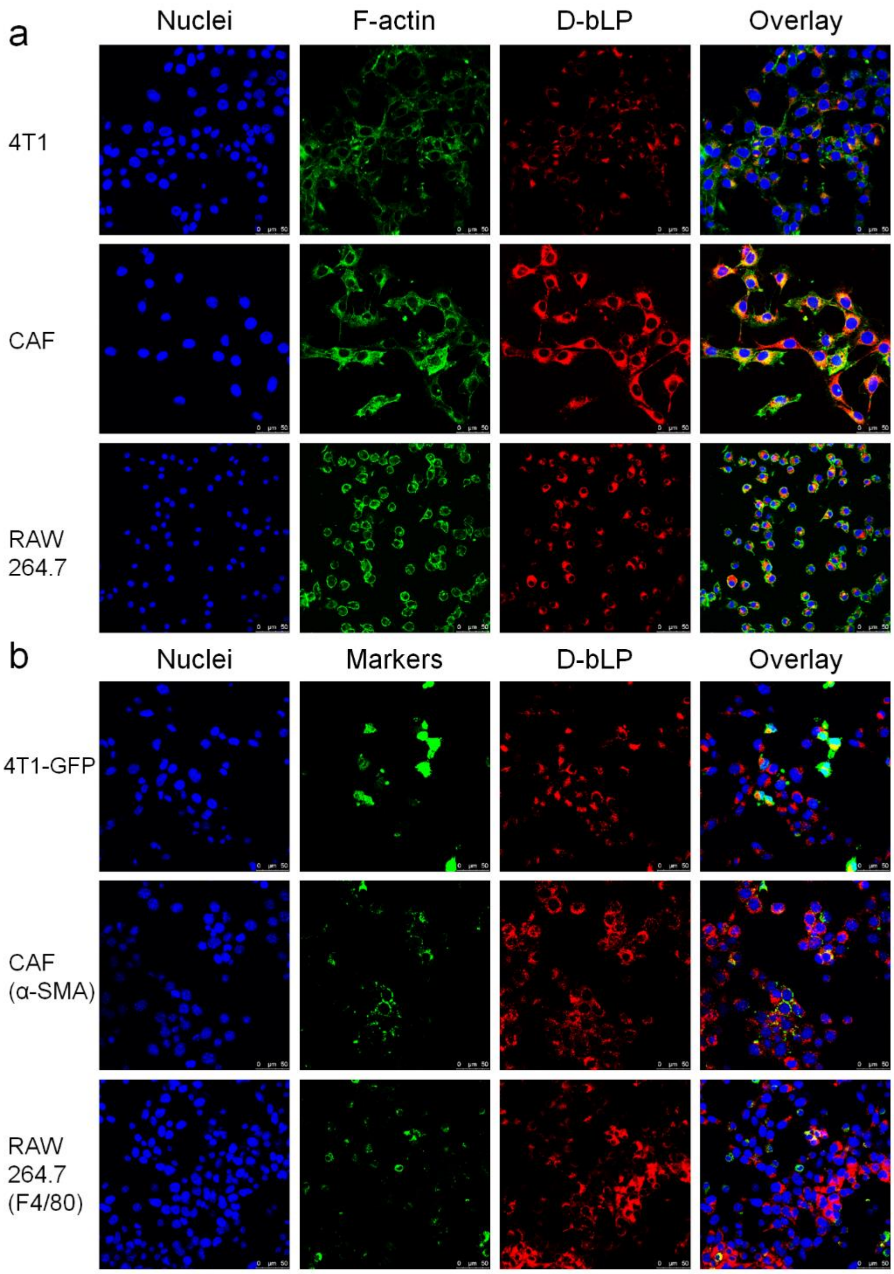

Supplementary Figure 22 Cellular uptake of D-bLP by 4T1 cancer cells, CAF and TAM (RAW 264.7) in single cell phenotype model and mixed cell phenotype model. **a** LCSM imaging of D-bLP in 4T1, isolated CAF and RAW 264.7, scale bar, 50 μm. **b** LCSM imaging of D-bLP in each cell phenotype from mixed cell model, scale bar, 50 μm. The 4T1 cancer cells, CAF and TAM in mixed cell model were respectively characterized as 4T1-GFP, α-SMA positive and F4/80 positive cells (green signals). The proportion of each cell phenotype in the mixed cell model was determined by flow cytometer analysis.

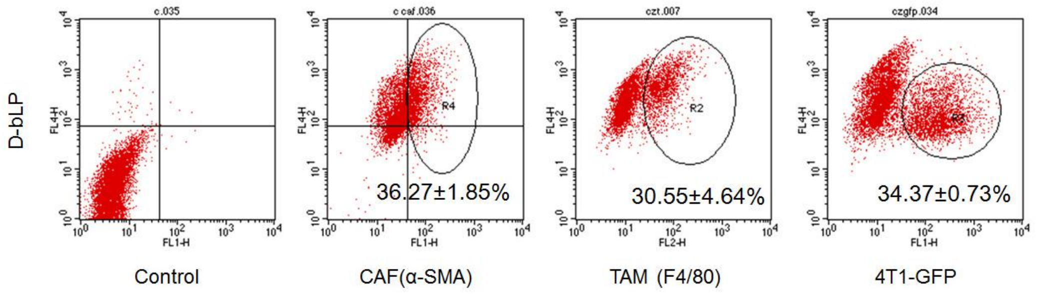

Supplementary Figure S23 The proportion of each cell phenotype in the mixed cell model of 4T1-GFP cancer cells, CAF and TAM (RAW 264.7), which was determined by flow cytometer analysis. Each cell phenotype of 4T1 cancer cells, CAF and TAM was respectively characterized as 4T1-GFP, α-SMA positive and F4/80 positive cells (n=3).

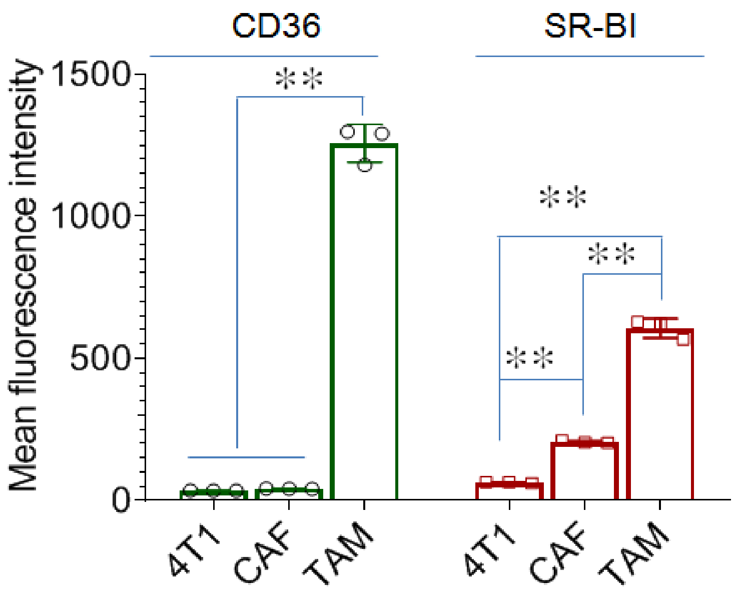

Supplementary Figure 24 The expression of SR-BI and CD36 in 4T1 cancer cells, CAF and TAM (RAW 264.7), which was determined by flow cytometer analysis. **a** Gate of each cell phenotype in flow cytometer analysis. **b** Quantified expression of CD36 and SR-BI in each cell phenotype. Data are means ± SD, n=3, **p<0.01 (ANOVA and two-tailed Student's t-test).

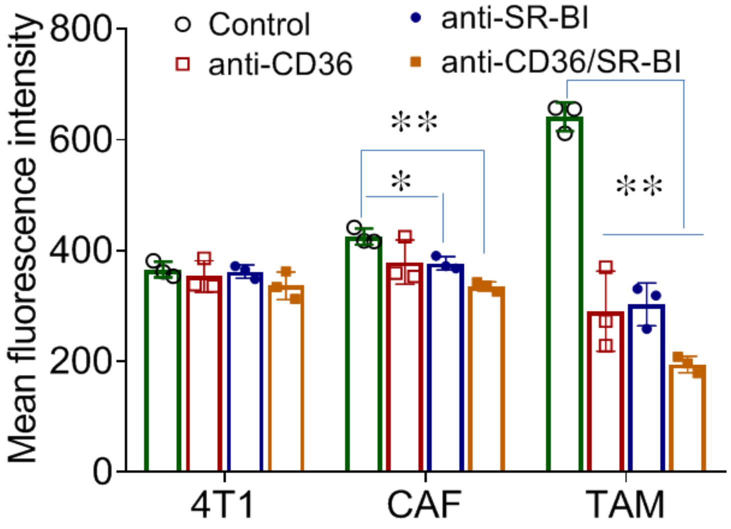

Supplementary Figure 25 Cellular uptake of D-bLP in 4T1 cancer cells, CAF and TAM (RAW 264.7) to investigate the possible mechanism of their preferential uptake by stromal cells. **a** Gate of each cell phenotype in flow cytometer analysis. **b** Quantified cellular uptake in each cell phenotype by flow cytometer analysis. Cells were respectively pretreated with specific antibodies of CD36, SR-BI, their combinations or absence (control), and then incubated with D-bLP for 4 h to evaluate their cellular uptake by flow cytometer analysis. Data are means ± SD, n=3, * $p<0.05$, ** $p<0.01$ (ANOVA and two-tailed Student's t-test).

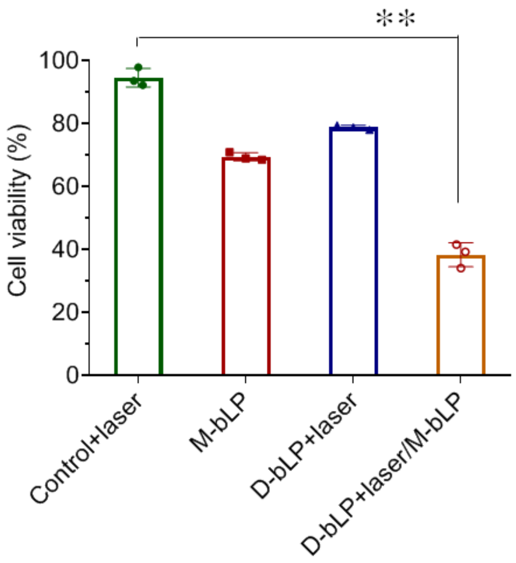

Supplementary Figure 26 The viability of metastatic 4T1 cancer cells treated with laser, D-bLP+laser (10 μg·mL^{-1} of DiR), M-bLP (1 μg·mL^{-1} of mertansine) and their combination of D-bLP+laser/M-bLP. Cells without any treatment were performed as negative control. Data are means ± SD, n=3, ** p<0.01 (two-tailed Student's t-test).

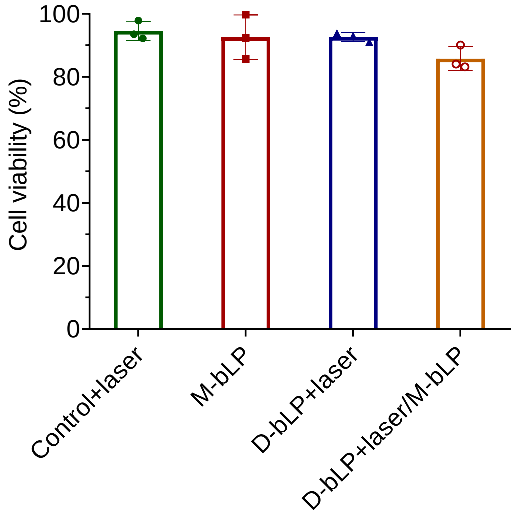

Supplementary Figure 27 The viability of metastatic 4T1 cancer cells treated with laser, D-bLP+laser (0.1 μg·mL^{-1} of DiR), M-bLP (0.01 μg·mL^{-1} of mertansine) and their combination of D-bLP+laser/M-bLP. Cells without any treatment were performed as negative control (n=3). Data are means ± SD.

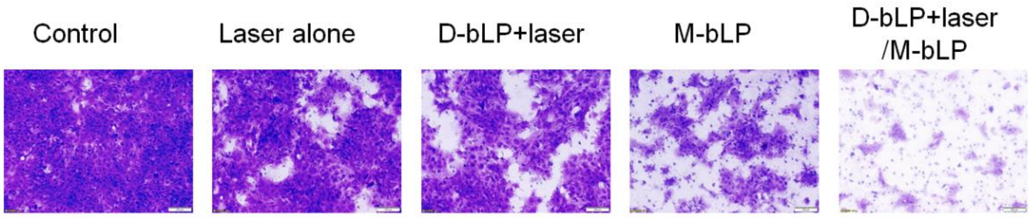

Supplementary Figure 28 The typical images of migrated 4T1 cells across the transwell membrane, wherein cells were pre-treated with laser, D-bLP+laser (0.1 μg·mL^{-1} of DiR), M-bLP (0.01 μg·mL^{-1} of mertansine) and their combination of D-bLP+laser/M-bLP. Cells without any treatment were performed as negative control, scale bar, 100 μm.

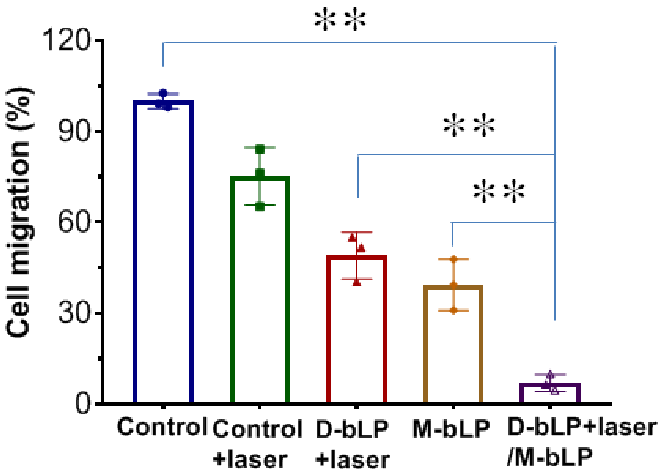

Supplementary Figure S29 The quantified migration of 4T1 cancer cells across the transwell membrane from each group. Cells were pre-treated with laser, D-bLP+laser (0.1 μg·mL^{-1} of DiR), M-bLP (0.01 μg·mL^{-1} of mertansine) and their combination of D-bLP+laser/M-bLP. Cells without any treatment were performed as negative control. Data are means ± SD, n=3, ** $p<0.01$ (ANOVA and two-tailed Student's t-test).

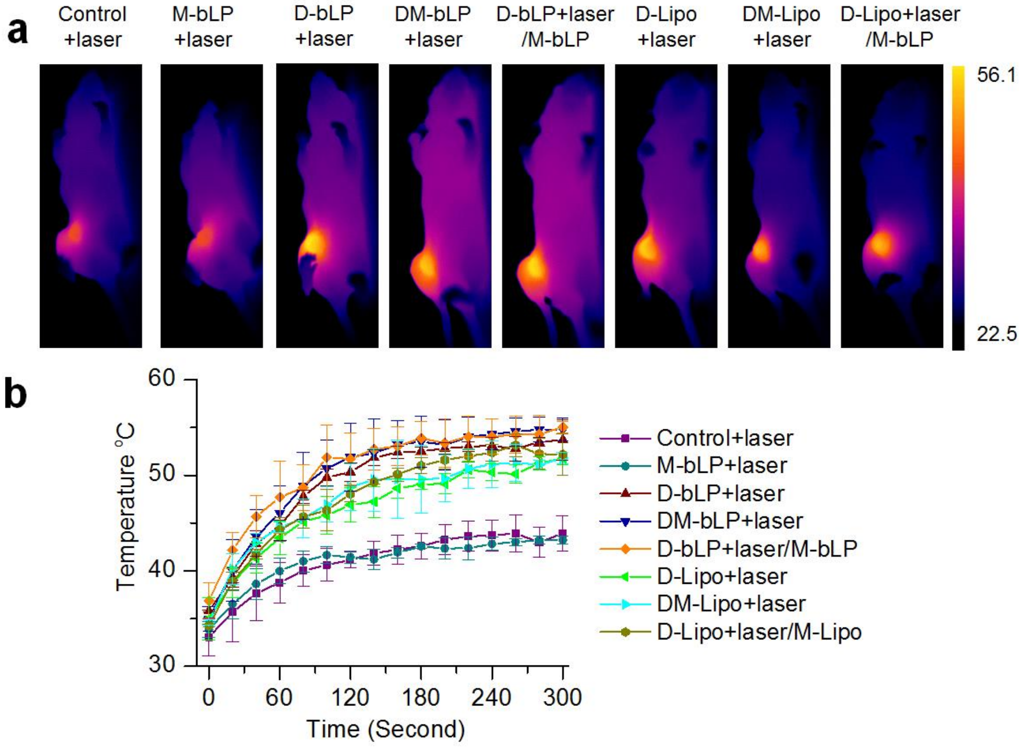

Supplementary Figure 30 The thermal profiles from each laser-irradiated group. **a** Typical thermal images of tumor bearing mice from each laser-irradiated groups. **b** Temperature changes of tumors from each laser-irradiated group. For control+laser and M-bLP+laser groups, n=4; other groups, n=3.

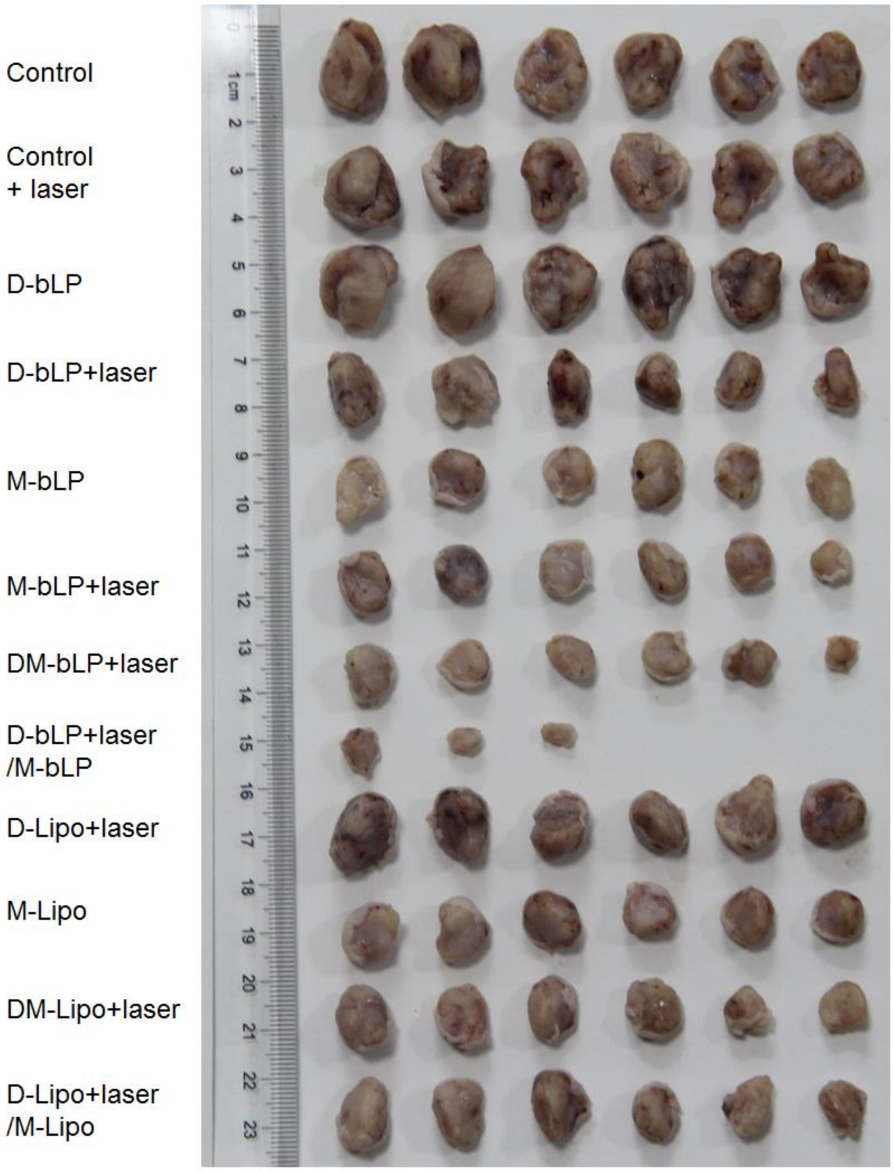

Supplementary Figure 31 The typical images of tumor tissues from each treatment (n=6). The tumor relapse was only detected in 3 of 6 mice in D-bLP+laser/M-bLP group.

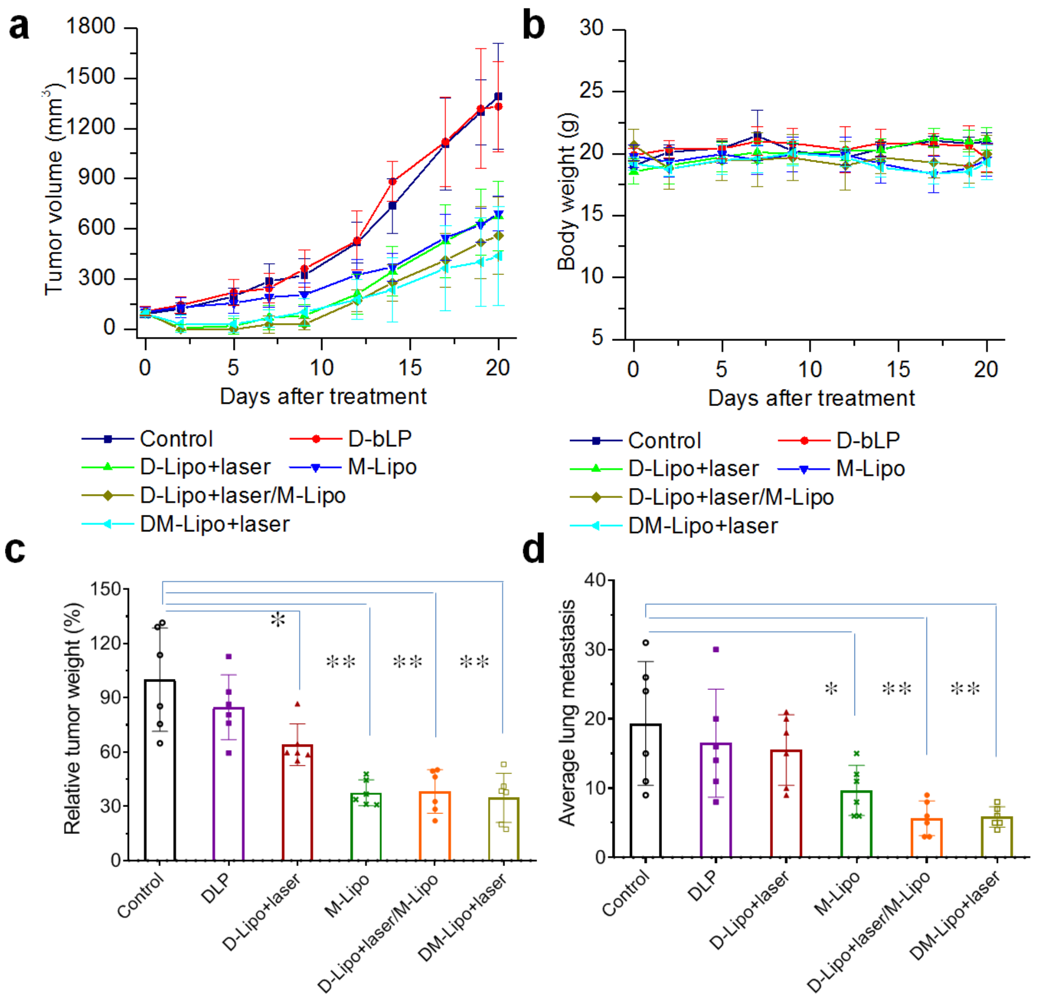

Supplementary Figure 32 The therapeutic effects of liposomes-based nanosystem on tumor growth and lung metastasis in 4T1-induced metastatic tumor model (n=6). **a** Tumor growth profiles from each group. **b** Body weight changes during these treatments. **c** Relative tumor weight from each group. **d** Average number of lung metastatic nodules from each group. Data are means ± SD, n=6, * $p<0.05$, ** $p<0.01$ (ANOVA and two-tailed Student's t-test).

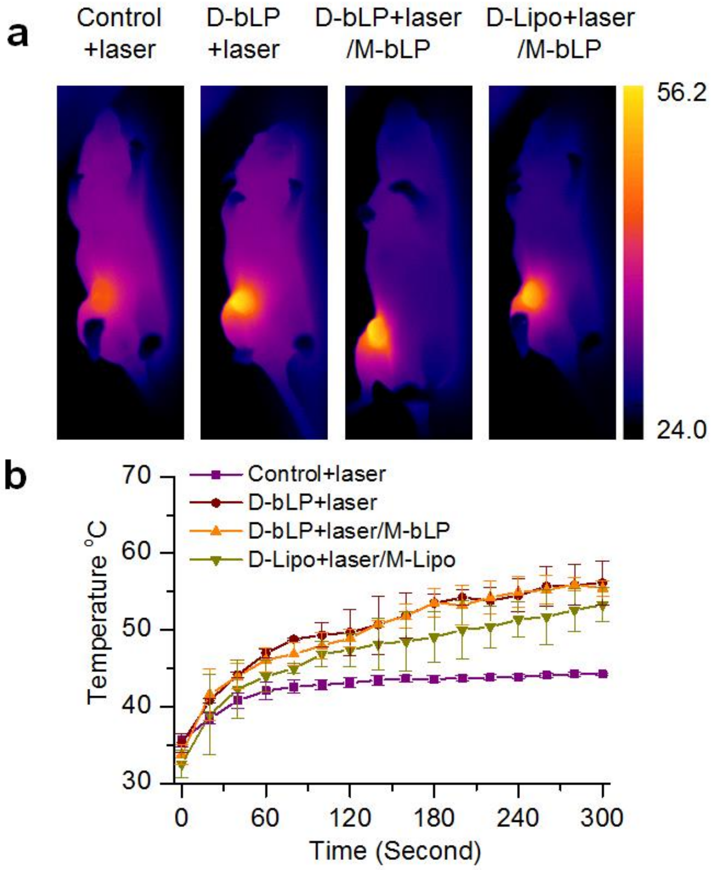

Supplementary Figure 33 The thermal profiles from each laser-irradiated group in MCF-7 induced breast cancer model. **a** Typical thermal images of tumor bearing mice from each laser-irradiated groups. **b** The temperature changes of tumors from each laser-irradiated groups. Data are means ± SD, n=3.

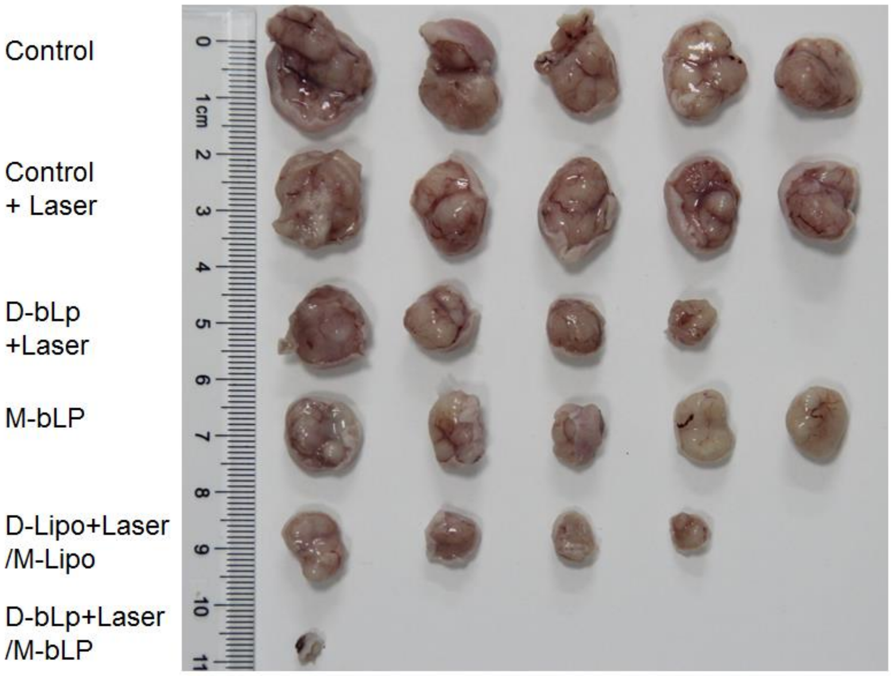

Supplementary Figure 34 Typical images of tumor mass from each group in MCF-7 induced tumor model. The tumor relapse was merely detected in 1 of 5 mice of D-bLP+laser/M-bLP group.

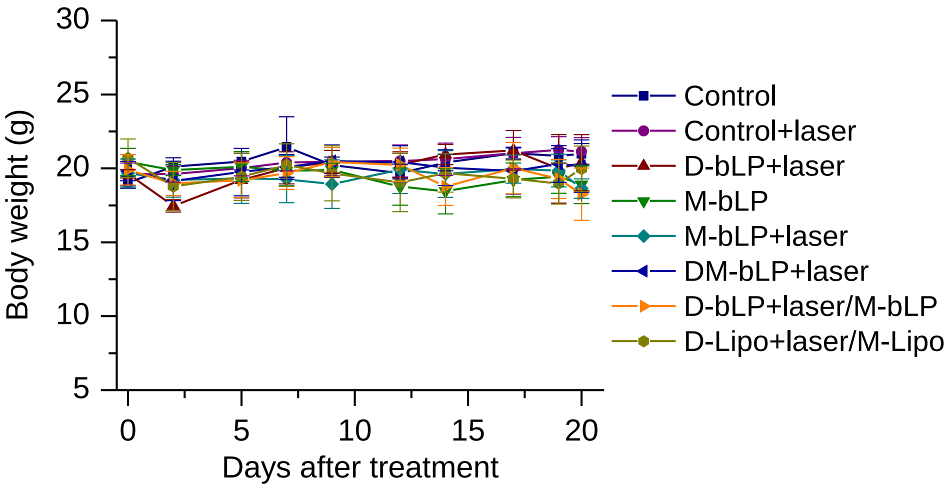

Supplementary Figure 35 The body weight changes in 4T1-induced tumor model from each group (n=6).

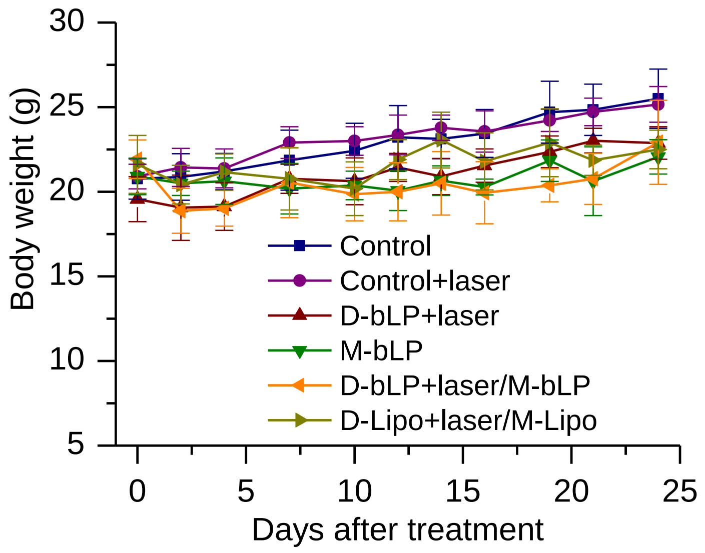

Supplementary Figure 36 The body weight changes of MCF-7 induced tumor model from each group (n=5).

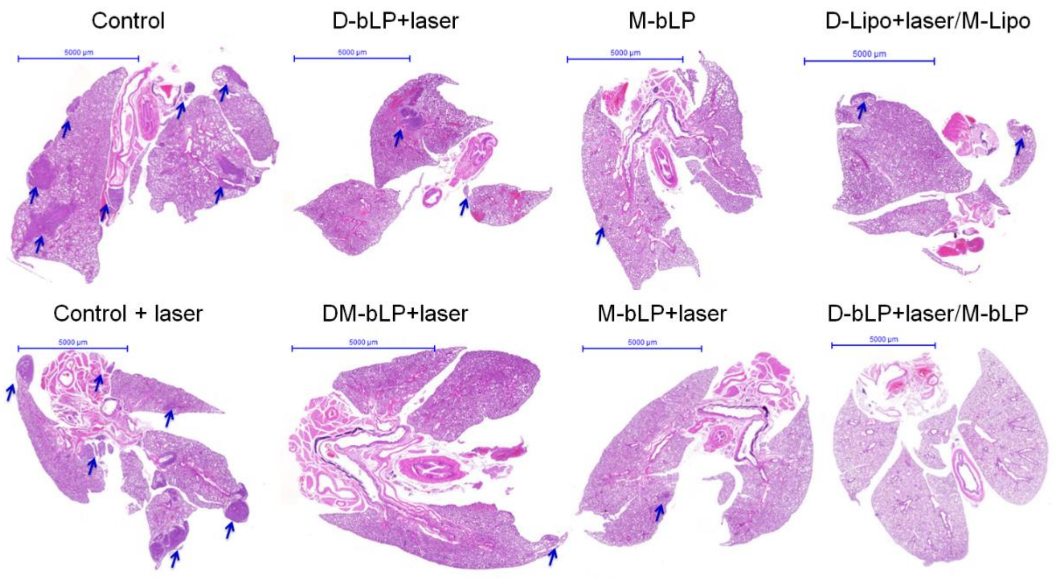

Supplementary Figure 37 Histological examination of the lung tissues from each group, which was determined by HE staining method, scale bar, 5000 μm. The metastatic nodules were presented as cell clusters with darkly stained nuclei (blue arrows).

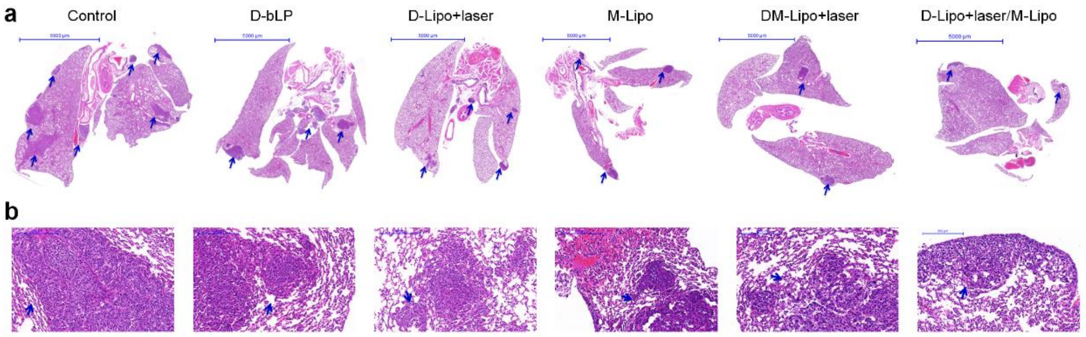

Supplementary Figure 38 Histological examination of lung tissues from each group in 4T1 induced tumor model, which was determined by HE staining method. **a** The images of the whole lung tissues, scale bar, 5000 μm. **b** The enlarged images of lung tissues with metastatic lesions, scale bar, 200 μm. The metastatic nodules were presented as cell clusters with darkly stained nuclei (blue arrows).

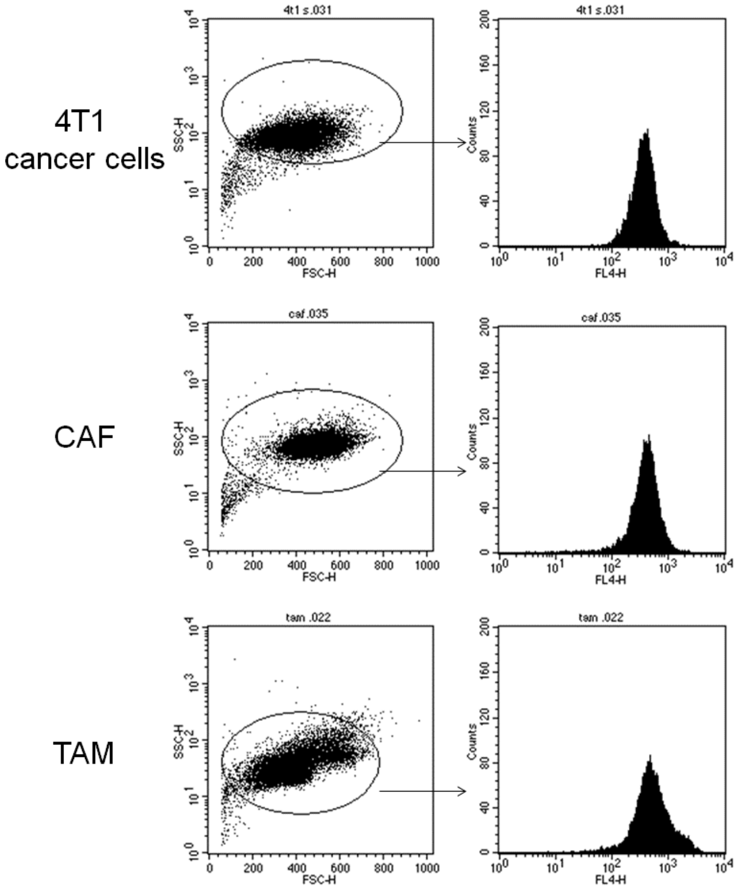

Supplementary Figure 39. Gating strategies used for flow cytometer analysis of single cell phenotype model of 4T1, isolated CAF and RAW 264.7 (TAM).

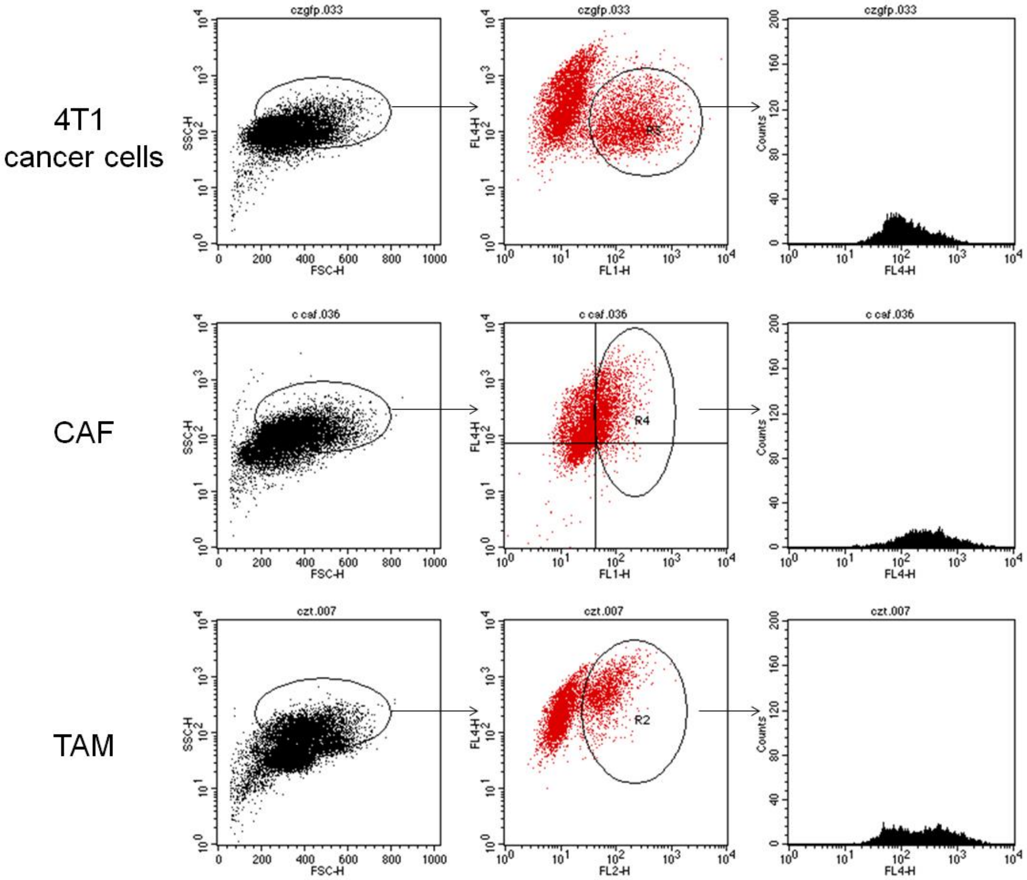

Supplementary Figure 40. Gating strategies used for flow cytometer analysis of mixed cell phenotype model of 4T1, isolated CAF and RAW 264.7 (TAM).

Supplementary Tables

Ingredients (%, w/w)	D-bLP	M-bLP	DM-bLP	Natural mature HDL
DiR	4.44±0.21	n.d.	4.80±0.27	n.d.
Mertansine	n.d.	0.464±0.004	0.390±0.005	n.d.
Peptide or Proteins	18.52±0.15	16.61±0.02	16.27±0.05	47.68±4.03
Phospholipids	77.04±4.82	82.93±6.19	79.21±0.34	27.04±3.53
Total cholesterol	n.d.	n.d.	n.d.	25.30±0.28
Triglyceride	n.d.	n.d.	n.d.	4.20±1.11
Zeta potential (mv)	+2.12±0.34	-4.95±0.73	+1.58±0.88	-9.91±0.93

Supplementary Table 1 The compositions and characteristics of each nanosystem including D-bLP, M-bLP, DM-bLP and natural mature HDL from human plasma. Data were presented as mean ± SD (n=3), n.d., no data. The phospholipids in these formulations were measured using the phospholipids assay kit (MAK122, Sigma-Aldrich). In natural mature HDL (Sigma, L1567), the total cholesterol was determined using the HDL quantitation kit (MAK045, Sigma-Aldrich), the triglyceride (TG) was quantified using the TG quantitation kit (MS2408, Shanghai Cablebridge Biotechnology Co. Ltd), and the protein content was measured using the protein assay kit (P0010, Beyotime). The content of ApoA1 mimetic peptide in re-constructed lipoproteins was analyzed using the UV-Vis spectroscopy. The drug loading capacity of bLP was presented as the percentage of entrapped DiR or mertansine in bLP compared to the total amount of all ingredients in D-bLP or M-bLP formulations.

Name	4T1	MCF-7
Control	6/6	5/5
Control+laser	6/6	5/5
D-bLP	6/6	n.d.
D-bLP+laser	6/6	4/5
M-bLP	6/6	5/5
M-bLP+laser	6/6	n.d.
DM-bLP+laser	6/6	n.d.
D-bLP+laser/M-bLP	3/6	1/5
D-Lipo+laser	6/6	n.d.
M-Lipo	6/6	n.d.
DM-Lipo+laser	6/6	n.d.
D-Lipo+laser/M-Lipo	6/6	4/5

Supplementary Table 2 The tumor relapse in 4T1 and MCF-7 induced orthotopic tumor models after each treatment, n.d., no data. The D-bLP+laser/M-bLP treatment produced efficient inhibition on tumor relapse, which was superior to other groups.

Groups	Number of metastatic nodules in each lung						Mean	SD
Control	31	24	26	15	11	9	19.3	8.9
Control+laser	30	22	16	15	9	11	17.2	7.7
D-bLP	30	20	11	16	8	14	16.5	7.8
D-bLP+laser	12	11	17	15	12	11	13	2.4
M-bLP	5	6	8	3	8	4	5.7	2.1
M-bLP+laser	4	6	7	5	2	9	5.5	2.4
DM-bLP+laser	3	1	3	2	2	4	2.5	1.0
D-bLP+laser/M-bLP	0	0	0	0	2	1	0.5	0.8
D-Lipo+laser	15	20	9	21	18	10	15.5	5.1
M-Lipo	15	8	6	12	11	6	9.7	3.6
DM-Lipo+laser	5	6	8	7	5	4	5.8	1.5
D-Lipo+laser/M-Lipo	9	6	3	8	5	3	5.7	2.5

Supplementary Table 3 The number of visualized metastatic nodules in lungs from 4T1 induced metastatic breast tumor model treated with 12 groups. The incidence of lung metastasis was detected in only 2 of 6 mice from D-bLP+laser/M-bLP group. The D-bLP+laser/M-bLP treatment resulted in a 97.4% inhibition of lung metastasis, which is more effective than other groups.
