# Supplementary material for: Bioinspired lipoproteins-mediated photothermia remodels tumor stroma to improve cancer cell accessibility of second nanoparticles
Source: Nat Commun. 2019 Jul 25;10:3322. doi: 10.1038/s41467-019-11235-4 (PMC6658501; doi:10.1038/s41467-019-11235-4)
Supplement: Supplementary file 2 — Reporting Summary [file 41467_2019_11235_MOESM2_ESM.pdf]

nature research

Corresponding author(s): Yaping Li

Last updated by author(s): Jun 17, 2019

Reporting Summary

Nature Research wishes to improve the reproducibility of the work that we publish. This form provides structure for consistency and transparency in reporting. For further information on Nature Research policies, see Authors & Referees and the Editorial Policy Checklist.

Statistics

For all statistical analyses, confirm that the following items are present in the figure legend, table legend, main text, or Methods section.

n/a	Confirmed	
☐	☒	The exact sample size (*n*) for each experimental group/condition, given as a discrete number and unit of measurement
☐	☒	A statement on whether measurements were taken from distinct samples or whether the same sample was measured repeatedly
☐	☒	The statistical test(s) used AND whether they are one- or two-sided<br>*Only common tests should be described solely by name; describe more complex techniques in the Methods section.*
☐	☒	A description of all covariates tested
☐	☒	A description of any assumptions or corrections, such as tests of normality and adjustment for multiple comparisons
☐	☒	A full description of the statistical parameters including central tendency (e.g. means) or other basic estimates (e.g. regression coefficient) AND variation (e.g. standard deviation) or associated estimates of uncertainty (e.g. confidence intervals)
☒	☐	For null hypothesis testing, the test statistic (e.g. *F*, *t*, *r*) with confidence intervals, effect sizes, degrees of freedom and *P* value noted<br>*Give P values as exact values whenever suitable.*
☒	☐	For Bayesian analysis, information on the choice of priors and Markov chain Monte Carlo settings
☒	☐	For hierarchical and complex designs, identification of the appropriate level for tests and full reporting of outcomes
☒	☐	Estimates of effect sizes (e.g. Cohen's *d*, Pearson's *r*), indicating how they were calculated

Our web collection on statistics for biologists contains articles on many of the points above.

Software and code

Policy information about availability of computer code

Data collection	Flow cytometry data ware collected using the BD CellQuest Pro. Other data were collected and analyzed using the Excel.
Data analysis	Excel/Origin 8.5/Graphpad prism 8

For manuscripts utilizing custom algorithms or software that are central to the research but not yet described in published literature, software must be made available to editors/reviewers. We strongly encourage code deposition in a community repository (e.g. GitHub). See the Nature Research guidelines for submitting code & software for further information.

Data

Policy information about availability of data

All manuscripts must include a data availability statement. This statement should provide the following information, where applicable:

- Accession codes, unique identifiers, or web links for publicly available datasets
- A list of figures that have associated raw data
- A description of any restrictions on data availability

The data supporting the findings of this study are available within this paper and Supplementary information. Source data of Fig 2c-e, 3c, 4e-f, 5d, 6c, 7e, 8c-e, 9b-f and Supplementary Figs 2, 3, 14, 20, 24-27, 29, 30b, 32 a-d, 33, 35, 36 are provided as a Source data file. Additional data can also be available from the corresponding author on reasonable request.

Field-specific reporting

Please select the one below that is the best fit for your research. If you are not sure, read the appropriate sections before making your selection.

☒ Life sciences ☐ Behavioural & social sciences ☐ Ecological, evolutionary & environmental sciences

For a reference copy of the document with all sections, see nature.com/documents/nr-reporting-summary-flat.pdf

Life sciences study design

All studies must disclose on these points even when the disclosure is negative.

Sample size	Sample size was determined by allowable error size and accuracy, which was detailed in the manuscript and supporting information.
Data exclusions	No data were excluded from the analyses.
Replication	Confirm the repeatability of the experimental findings.
Randomization	Samples were randomly grouped.
Blinding	The investigators were blinded to group allocation during data collection and analysis.

Reporting for specific materials, systems and methods

We require information from authors about some types of materials, experimental systems and methods used in many studies. Here, indicate whether each material, system or method listed is relevant to your study. If you are not sure if a list item applies to your research, read the appropriate section before selecting a response.

Materials & experimental systems

n/a	Involved in the study	
☐	☒	Antibodies
☐	☒	Eukaryotic cell lines
☒	☐	Palaeontology
☐	☒	Animals and other organisms
☒	☐	Human research participants
☒	☐	Clinical data

Methods

n/a	Involved in the study	
☒	☐	ChIP-seq
☐	☒	Flow cytometry
☒	☐	MRI-based neuroimaging

Antibodies

Antibodies used	All antibodies were detailed in the Methods with supplier names, catalog number and dilution folds, etc.
Validation	All antibodies are commercially available, and have been verified by suppliers.

Eukaryotic cell lines

Policy information about cell lines

Cell line source(s)	Mouse 4T1 , 4T1-GFP, RAW 264.7, Human MCF-7
Authentication	Mouse 4T1 cells, RAW 264.7 cells and human MCF-7 cells were provided from Cell Bank of Shanghai, Chinese Academy of Sciences. The 4T1-GFP cells were obtained from Keyuandi Biotech Co. Ltd (Shanghai, China).
Mycoplasma contamination	Negative for mycoplasma.
Commonly misidentified lines (See ICLAC register)	No misidentified lines.

Animals and other organisms

Policy information about studies involving animals; ARRIVE guidelines recommended for reporting animal research

Laboratory animals	Female Balb'c nude mice within 18-22 g, which is listed in the Methods section.
Wild animals	No wild animals were used.
Field-collected samples	No samples were collected from field.
Ethics oversight	The animal experiments were performed according to the protocols approved by the Institutional Animal Care and Use Committee (IACUC) of Shanghai Institute of Materia Medica, Chinese Academy of Science.

Note that full information on the approval of the study protocol must also be provided in the manuscript.

Flow Cytometry

Plots

Confirm that:

- ☒ The axis labels state the marker and fluorochrome used (e.g. CD4-FITC).
- ☒ The axis scales are clearly visible. Include numbers along axes only for bottom left plot of group (a 'group' is an analysis of identical markers).
- ☒ All plots are contour plots with outliers or pseudocolor plots.
- ☒ A numerical value for number of cells or percentage (with statistics) is provided.

Methodology

Sample preparation	The detailed information of samples preparation were listed in the Methods.
Instrument	BD FACS Calibur system
Software	BD CellQuest Pro.
Cell population abundance	No post-sort fractions were collected
Gating strategy	All experiments included an unstained sample and single-stained samples as compensation to define negative and positive cell populations. The preliminary gates excluded cell fragments. The gating strategies were listed in Fig 8g, Supplementary Fig 39, 40.

☒ Tick this box to confirm that a figure exemplifying the gating strategy is provided in the Supplementary Information.
